# Cognitive processing of orientation discrimination in anisometropic amblyopia

**DOI:** 10.1371/journal.pone.0186221

**Published:** 2017-10-12

**Authors:** Jianglan Wang, Jiao Zhao, Shoujing Wang, Rui Gong, Zhong Zheng, Longqian Liu

**Affiliations:** 1 Department of Optometry and Visual Science, West China School of Medicine, Sichuan University, Chengdu, Sichuan Province, China; 2 Department of Ophthalmology, People’s Hospital of Leshan, Leshan, Sichuan Province, China; 3 Medical Engineering Department, West China Hospital, Sichuan University, Chengdu, Sichuan Province, China; 4 Department of Ophthalmology, West China Hospital, Sichuan University, Chengdu, Sichuan Province, China; 5 Neurobiological Laboratory Center, West China Hospital, Sichuan University, Chengdu, Sichuan Province, China; University Medical Center Goettingen, GERMANY

## Abstract

Cognition is very important in our daily life. However, amblyopia has abnormal visual cognition. Physiological changes of the brain during processes of cognition could be reflected with ERPs. So the purpose of this study was to investigate the speed and the capacity of resource allocation in visual cognitive processing in orientation discrimination task during monocular and binocular viewing conditions of amblyopia and normal control as well as the corresponding eyes of the two groups with ERPs. We also sought to investigate whether the speed and the capacity of resource allocation in visual cognitive processing vary with target stimuli at different spatial frequencies (3, 6 and 9 cpd) in amblyopia and normal control as well as between the corresponding eyes of the two groups. Fifteen mild to moderate anisometropic amblyopes and ten normal controls were recruited. Three-stimulus oddball paradigms of three different spatial frequency orientation discrimination tasks were used in monocular and binocular conditions in amblyopes and normal controls to elicit event-related potentials (ERPs). Accuracy (ACC), reaction time (RT), the latency of novelty P300 and P3b, and the amplitude of novelty P300 and P3b were measured. Results showed that RT was longer in the amblyopic eye than in both eyes of amblyopia and non-dominant eye in control. Novelty P300 amplitude was largest in the amblyopic eye, followed by the fellow eye, and smallest in both eyes of amblyopia. Novelty P300 amplitude was larger in the amblyopic eye than non-dominant eye and was larger in fellow eye than dominant eye. P3b latency was longer in the amblyopic eye than in the fellow eye, both eyes of amblyopia and non-dominant eye of control. P3b latency was not associated with RT in amblyopia. Neural responses of the amblyopic eye are abnormal at the middle and late stages of cognitive processing, indicating that the amblyopic eye needs to spend more time or integrate more resources to process the same visual task. Fellow eye and both eyes in amblyopia are slightly different from the dominant eye and both eyes in normal control at the middle and late stages of cognitive processing. Meanwhile, abnormal extents of amblyopic eye do not vary with three different spatial frequencies used in our study.

## Introduction

Amblyopia is a neural-developmental visual disorder without obvious organic deficits caused by inadequate early visual experience. It is always accompanied by one or more known factors, such as anisometropia (anisometropic amblyopia), strabismus (strabismic amblyopia), high refractive error (refractive error amblyopia), ptosis and cataract (form-deprivation amblyopia) [1, 2]. Anisometropic amblyopia is very common one [2]. The prevalence of amblyopia is approximately 1–3% in humans [3]. Amblyopia is associated with reduced spatiotemporal vision that affects visual acuity[4, 5], vernier acuity [6], contrast sensitivity [7], stereopsis [8], and abnormal spatial interactions [9].

Previous psychophysical and neuroimaging studies have suggested that amblyopia not only leads to abnormal responses in the primary and secondary visual areas but also deficits at higher levels of visual pathways [10]. In addition, some studies have observed abnormal neural responses in subjects with amblyopia using electrophysiological techniques [11–14]. However, the impairments in amblyopia are not completely understood.

Now we could use event-related potentials (ERPs) with high temporal resolution to explore this question because ERPs could reflect the physiological changes of the brain during the processes of cognition such as attention, memory, thinking[15, 16]. P300, a later component of ERPs, is a positive wave recorded between a 300–600 ms time window after stimulus onset. P300 refers to the middle and late stages of cognitive processing that occur prior to the selection and preparation of motor responses and can be used to measure cognitive capability [17, 18]. The reliability of P300 measurements is comparable to clinical assays, and these values can be attained inexpensively [19]. As a result, P300 has been widely used in cognitive studies of various diseases [20–22]. Because amblyopia presents with visual cognition deficits such as abnormal visual integration [23, 24] and motion perception [25, 26], we used P300 to investigate the speed and the capacity of resource allocation in cognitive processing of subjects in monocular and binocular conditions of amblyopia and normal control as well as between the corresponding eye of two groups. Explore the associated neural mechanisms through this method. The corresponding eyes of two groups were amblyopic eye and non-dominant eye, fellow eye and dominant eye, or binoculus in amblyopia and binoculus in normal control respectively.

P300 can be elicited reliably through an oddball paradigm using a variety of stimuli, such as visual, auditory or sensory stimuli. P300 amplitude and latency, by assessing the processing capacity and speed, are linked to a variety of attentional and memory processes [27–29], and these measures have been successfully applied to discriminate abnormal from healthy subjects [30, 31]. Generic P300 consists of two subcomponents, P3a and P3b, which represent distinct but related neural processes. These components can be elicited separately by specific stimuli and task conditions [32]. ‘Novel’ stimuli as infrequent non-target stimuli can generate novelty P300, a kind of P3a. Novelty P300, related to the orienting response, generally exhibits a frontal scalp distribution, has a relatively short latency, and habituates rapidly. Because novelty P300 is thought to reflect frontal lobe function, its amplitude can indicate attentional orienting with increased amplitude related to greater focal attention [33, 34].

The P3b component is elicited by target stimuli with maximum amplitude over the parietal cortex [35]. The amplitude of P3b is determined by the allocation of attentional resources due to updated working memory [17, 36]. P3b latency, often independent of response selection and behavioral action [37, 38], is generally considered to represent the speed of stimulus evaluation and classification [39] and can be used as a measure of stimulus detection and evaluation time [40]. P3a is generated when a demanding stimulus commands frontal lobe attention; P3b is generated when memory updating in the associated cortex requires an allocation of attentional resources [41].

It remains unknown how visual cognition processing is conducted in monocular and binocular viewing conditions in amblyopia and normal control as well as between the corresponding eyes of two groups at different spatial frequencies. In order to investigate these questions, we explored cognition processing with novelty P300 and P3b using ERPs.

A three-stimulus oddball paradigm (target, novel, and standard stimuli) was used with Gabor patch orientation discrimination tasks with low, medium, and high spatial frequencies (3, 6 and 9 cycle per degree, cpd, respectively) in monocular and binocular conditions. To explore whether the P300 latency is affected by previous components latency, N2 latency is measured and analyzed. If N2 latency is the same in different eye conditions and the corresponding eyes of two groups, it shows that the longer P300 latency does not result from the longer previous components latency or otherwise. The time window was 200–400 ms for N2, 250–550 ms for novelty P300, and 300–600 ms for P3b.

If the decreased ACC, the longer RT the longer latency or larger amplitude of Novelty P300 or P3b in amblyopic eye compared with fellow eye, both eyes of amblyopia or non-dominant eye of normal control, but these parameters are consistent in different eye conditions of normal control. The result may imply that the cognitive process of amblyopic eye is abnormal. In addition, if these parameters of the fellow eye and both eyes of amblyopia are different with their corresponding eyes of normal control, it suggests that the cognitive process is inconsistent between the corresponding eyes of two groups.

## Materials and methods

### Subjects

Anisometropic amblyopes and normal controls were included from the outpatient department of West China Hospital of Sichuan University from July 1st in 2015 to April 31st in 2016. The experiment, a part of “Investigations of Visual Cortex Defects In Strabismus and Amblyopia”, was in accordance with the Declaration of Helsinkiand and conducted after obtaining the approval from the institutional review board of West China Hospital of Sichuan [No 2014 (33), 1-6-2015]. All the parents of children and adult participants gave their informed and written consents to participate in the study before testing began. All subjects were examined by the same ophthalmologist in West China Hospital of Sichuan University. The procedures were as follows. First, the health of outer eye, anterior eye, and fundus were checked. The outer eye and anterior eye were checked with slit lamp (LS-6, Shangbang Medical Instrument. Co., Ltd. Chongqing, China), and fundus with both direct ophthalmoscope (Medtrue Enterprise Co., Ltd. Jiangsu, China) and Spectralis Optical Coherence Tomography (Heidelberg Engineering, Dossenheim, Germany). Then, refraction was tested under cycloplegics to get more accurate outcome. Finally, visual function, such as vision, near stereopsis, was tested under optimal corrections of spectacles. Vision was measured with ETDRS vision chart (Precision Vision, IL, US) with the distance of 4 meters. Amblyopic eye or non-dominant eye was tested firstly with the unused covered, and then the fellow eye or dominant eye was measured with another eye covered, and the both eyes were tested at last in two groups. The measurement was recorded with LogMAR acuity. Near stereopsis was tested with random dot (total 10 levels from 20 to 400 arc sec) at 40 cm (Vision Assessment Corporation, USA) when the subjects wore their best corrected spectacles with the polarized glasses outside.

Inclusion criteria for anisometropic amblyopia were the following: best corrected LogMAR visual acuity of the amblyopic eye (AE) in the range of 0.2 to 0.5, without any ocular organic abnormalities (except for refraction error), and 0 or better in the fellow eye (FE). Inclusion criteria for normal control were the best corrected LogMAR acuity of each eye was 0 or better. The dominant eye was decided with the hole-in-the-card test [42, 43]. All participants were right-handed and without significant physical or mental illnesses. Handedness was assessed with a standard handedness questionnaire [44].

### Visual stimuli and procedures

In the formal tests, the participant sat in a quiet room with soft lighting. All stimuli and the black fixation cross were presented centrally on a gray background with a luminance of 36cd/m^2^ measured with luminance meter. The participant sat in a comfortable position at a distance of 100 cm to the screen. The subject placed his/her chin on a chin rest and viewed the central display horizontally and then performed the orientation discrimination task.

**Fig 1:**
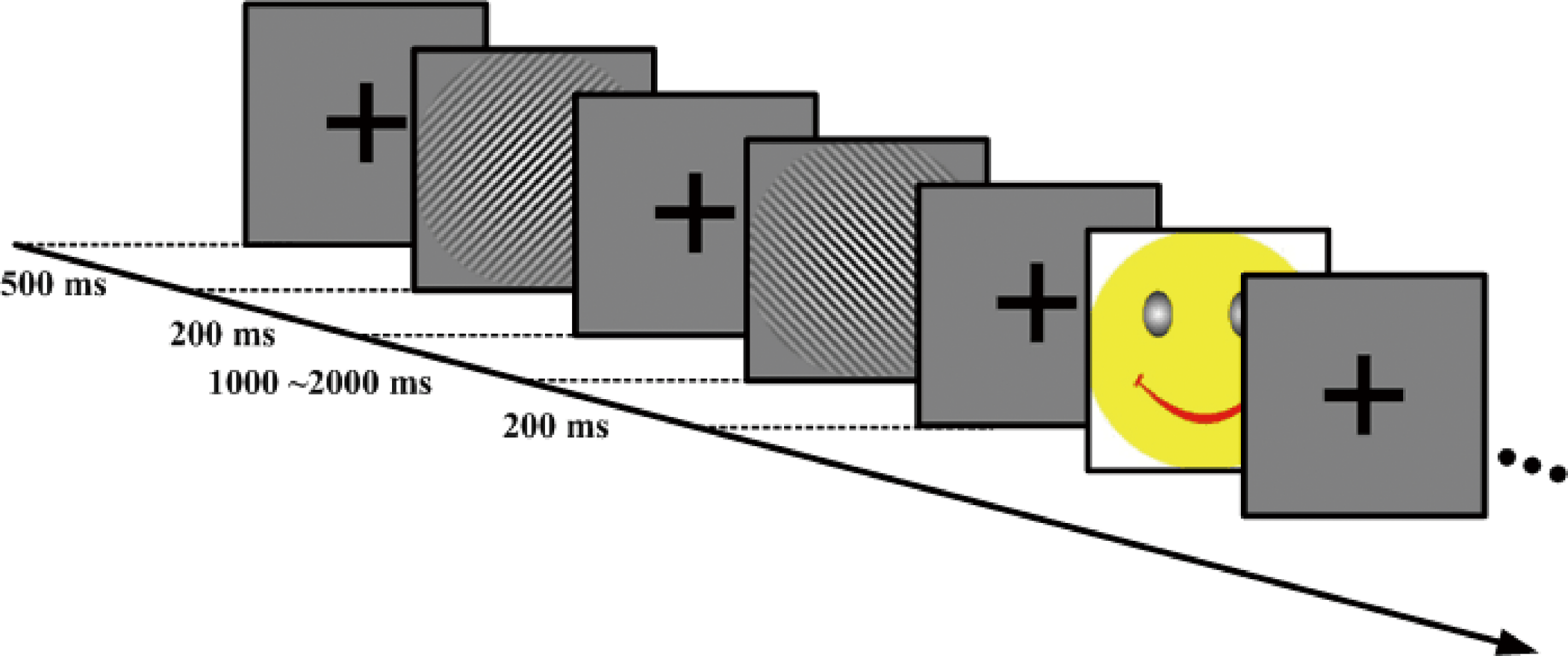
Experimental protocol showing the general stimulus sequence (three trials). The sequence of stimuli for a given trial was as follows. First, a black "+" was presented for 500 ms on a gray screen, followed by a stimulus for 200 ms. Next, the participant was asked to discriminate the orientation of 135 degrees Gabor patches (target stimuli) by pressing the Enter key as fast and accurately as possible. Subsequently, after stimulus presentation, a gray screen with a central black “+” was randomly presented for 1,000–2,000 ms, followed by a stimulus of 200 ms.

Three different images (45 degrees oriented and 135 degrees oriented Gabor patch with 0.5 degrees half Gaussian ramp in the periphery area, and smiling face) were randomly presented at the center of a 26” Dell LCD monitor with 1024×768 pixel resolution, and a refresh rate of 60 Hz. The participants performed an orientation discrimination task (Fig 1). All Gabor patches were sine-wave gratings with a contrast sensitivity of 98% to ensure the accuracy (ACC) was no less than 80% for all subjects. All stimuli subtended 9 degrees×9 degrees in size at the testing distance of 100 cm in every block. Low, medium and high spatial frequency (3, 6 and 9 cpd) were tested respectively. Each block lasted approximately 6 minutes and included 200 trials, of which 70% were 45 degrees oriented Gabor patch (standard stimuli), 20% were 135 degrees oriented Gabor patch (target stimuli) and 10% were smiling faces (novel stimuli). Each stimulus was presented for 200 ms, and the interval between successive stimuli onsets randomly varied between 1,000 ms and 2,000 ms. A black cross (0.5 degrees×0.5 degrees) was continuously visible at the center of the display during the interval to keep the subjects’ eyes fixated. Eye movements were monitored with Eyelink-1000 (SR Research Ltd, Ontario, Canada) in order to confirm the subject fixate the cross during the test. Observers were required to press the Enter key on a keyboard as soon as the target stimulus was presented.

The maximum response duration allowed was 1,000 ms. Each participant took a 5-minute break after one block of the trials. Every participant performed nine blocks (3 spatial frequencies ×3 eye conditions). The amblyopic eye (AE) and non-dominant eye (ND) were measured first, followed by the fellow eye (FE) and dominant eye (DE) when the unused eye was completely patched during the test. Finally, both eyes of amblyopia (BA) and control (BC) were tested. E-prime 2.0 software (PsychologySoftware Tools, Inc., Sharpsburg, PA) was used in the tests to present and record data. In the tests, a value of 1 was recorded when the response was identified correctly, and a value of 0 was recorded when a wrong answer was provided. After the test, accuracy (ACC) in percentage would be presented so that the subject knew whether he or she got the accuracy ≥80%. They had to retest until the accuracy was ≥80%. The reaction time (RT) from the presentation of the stimulus to when the key was pressed was recorded simultaneously. The participants placed the index finger of the right hand on the Enter key so that they could press it as soon as possible once the target stimuli were presented. Prior to the test, the investigator read the instructions to the participants to make sure they fully understood the experimental requirements. Participants then pressed the Enter key to begin the test. Each participant received a short practice session before the formal test to ensure that he or she understood how to operate the equipment successfully.

### Electrophysiological acquisition and processing

The acquisition and processing techniques of ERP were described by Banko[11]. Electroencephalographic (EEG) data were acquired using a BrainAmp MR amplifier system (Brain Products GmbH, Munich, Germany) and an elastic cap (Easycap GmBH, Herrsching-Breitbrunn, Germany) where 64 Ag/AgCI electrodes were mounted according to a modified 10–20 placement system. Meanwhile, one additional periocular electrode above the left eye was used to record the electrooculogram. All scalp resistances of electrodes were maintained below 10 KΩ. The sample rate was at 1000Hz and the bandpass filtering was 0.5-30Hz. Continuous EEG and behavioral data were simultaneously recorded. The baseline measure was subtracted 200ms from the signal prior to stimulus presentation. Artifacts were rejected and the baseline was corrected with the data in. the 1000ms epochs (-200 to 800 ms relative to stimulus). Data were processed using Brain Vision Analyzer 2.0 (Brain Products GmbH, Munich, Germany) off-line.

The waveforms (N2, novelty P300, and P3b) of each eye condition in two groups (anisometropic amblyopoes and normal controls) were superimposed to generate two types of ERP (target stimuli and novel stimuli) over three midline electrodes (FZ, CZ, PZ).

The amount of accessibly average waveforms was no less than 80% of total number. That is to say, the number of average waveforms in target stimuli is no less than 32 and 16 in novelty stimuli. The N2 components were defined as the largest negative-going peaks occurring within 200–400 ms occurring before Novelty P300 or P3b. The novelty P300 and P3b components were defined as the largest positive-going peaks occurring within 250–550 ms in novelty stimuli and 300–600 ms in target stimuli respectively. The amplitude was recorded as the difference between the mean pre-stimulus baseline and the maximum peak amplitude. The peak latency was measured as the time point corresponding to the maximum amplitude[41].

The mean latency and amplitude of novelty P300 and P3b and the mean latency of N2 in different stimulus conditions were subjected to statistical analyses.

### Statistical analysis

#### Demographic data analysis

Age between two groups was analyzed with independent sample t test. Analysis was performed with SPSS 20.0 (IBM Inc, Chicago, Illinois, USA), and *P* values less than 0.05 was considered statistically significant.

#### Behavioral data analysis

Behavioral data, ACC and RT were analyzed with 3 (eye conditions: amblyopic eye, fellow eye and both eyes in amblyopia group; non-dominant eye, dominant eye and both eyes in normal control group, respectively) × 3 (frequencies: 3, 6 and 9 cpd) univariate repeated measures ANOVA to compare whether the differences existed in different eye conditions and/or spatial frequencies in amblyopic group and normal control group, respectively. In addition, ACC and RT were analyzed with 2 (two groups: amblyopic eye and non-dominant eye, fellow eye and dominant eye, both eyes of amblyopia and both eyes of normal control) × 3 (frequencies: 3, 6 and 9 cpd) univariate repeated measures ANOVA to compare whether the differences existed in the group and/or spatial frequencies between the two groups. Post-hoc t-tests were computed using Tukey honest significant difference (HSD) tests. Analysis was performed with the same SPSS software and *P* values less than 0.05 was considered statistically significant.

#### ERP data analysis

Statistical analysis was performed on the averaged ERPs waveforms (N2, novelty P300, and P3b) over midline electrodes of three brain areas (F_Z_, C_Z_, P_Z_). The average waveforms were generated from the combined data of all relevant subjects. The mean latency and amplitude of novelty P300 and P3b but only the mean latency of N2 for the different stimulus conditions was subjected to the univariate repeated measures ANOVA. The specific methods would be presented in the relevant part. Post-hoc t-tests were computed using Tukey HSD tests. In addition, the latency of N2 between the corresponding eyes of two groups was analyzed with independent sample t test. The relationship between RT and P3b latency was analyzed with the Pearson correlation method. Analyses were performed with the same SPSS software and *P* values less than 0.05 was considered statistically significant.

## Results

### Demographic results

Fifteen anisometropic amblyopes (mean age ± SD: 18.3±3.6 years) and ten normal controls (mean age ± SD: 17.7±3.9 years) were recruited. There was no significant difference between two groups (*t*_23_ = 0.374, *P*>0.05). The clinical information is showed in Table 1.

**Table 1:** Clinical details of participants of anisometropic amblyopia and normal control.

Subject (Group)		Refraction		Visual Acuity (LogMAR)		
	Age/Sex	RE	LE	RE	LE	Stereopsis
1 (AA)	15/F	-1.00DS	+2.25DS	00	0.4	200"
2 (AA)	16/M	-0.50DS	+2.75DS/+1.00DC×90	00	0.5	400"
3 (AA)	14/F	+3.00DS/+0.75DC×85	-0.75DS/-0.75DC×10	0.4	00	400"
4 (AA)	20/M	PL	+1.75DS	0	0.3	200"
5 (AA)	22/F	-4.25DS	+2.50DS/+2.00DC×5	0	0.5	400"
6 (AA)	18/F	-2.00DS	+1.50DS/+1.50DC×90	-0.1	0.4	200"
7 (AA)	17/M	-1.75DS	+3.00DS/+0.50DC×85	0	0.3	160"
8 (AA)	18/M	-1.00DS	+2.50DS/+1.50DC×90	0	0.3	200"
9 (AA)	13/F	-0.75DS	+3.50DS/+1.00DC×90	0	0.5	200"
10 (AA)	16/M	-2.50DS	+4.00DS	-0.1–0.1	0.5	400"
11 (AA)	24/F	-3.50DS	+2.00DS/+1.50DC×80	0	0.3	200"
12 (AA)	16/F	-0.75DS	+2.75DS	0	0.3	200"
13 (AA)	23/F	+2.25DS/+0.50DC×80	-1.50DS	0.3	0	200"
14 (AA)	24/M	PL	+2.00DS/+2.00DC×90	0	0.4	200"
15 (AA)	18/F	-2.00DS	+3.50DS/+1.00DC×180	0	0.5	400"
16 (NC)	24/F	-6.00DS	-5.50DS/-0.75DC×160	0	0	40"
17 (NC)	16/F	-2.75DS/-1.50DC×170	-2.75DS/-1.75DC×170	0	0	20"
18 (NC)	20/F	-6.00DS/-1.50DC×90	-5.50DS	0	-0.1	40"
19 (NC)	20/F	-6.25DS/-1.00DC×164	-5.25DS/-1.00DC×170	-0.1	0	40"
20 (NC)	18/F	-1.00DS	PL	-0.1	0	40"
21(NC)	14/F	-2.75DS/-1.00DC×35	-4.00DS/-0.50DC×180	0	0	40"
22 (NC)	18/F	-3.00DS	-2.00DS/-1.00DC×5	-0.1	-0.1	40"
23 (NC)	22/M	PL	PL	-0.1	-0.1	20"
24 (NC)	21/F	-6.00DS/-1.00DC×180	-4.50DS/-1.75DC×180	-0.1	0	40"
25 (NC)	13/F	-1.75DS/-2.50DC×180	-0.50DS/-2.50DC×180	-0.1	-0.1	40"

AA: Anisometropic Amblyopia, NC: Normal Control, RE: Right Eye, LE: Left Eye, VA: Visual Acuity, M: Male, F: Female, PL: Plane lens, DS: Dioptric Sphere, DC: Dioptric Cylinder

### Behavioral results

The ACC and RT of the anisometropic amblyopia and normal control at three spatial frequencies tasks are shown in Table 2.

**Table 2:** The accuracy (ACC, %) and reaction time (RT, ms) of the anisometropic amblyopic group and normal controls in the orientation discrimination task.

Frequency	Eye	ACC(%)	RT(ms)
3 cpd	AE/ND	96.9±2.6 / 96.3±3.5	344.07±41.54 / 322.72±71.26
	FE/DE	97.1±2.1 / 96.5±6.3	345.00±49.27 / 332.95±69.57
	BA/BC	95.9±4.2 / 96.6±6.1	316.46±44.74 / 318.69±85.87
6 cpd	AE/ND	97.1±2.1 / 95.2±6.3	363.39±37.67 / 325.40±67.14
	FE/DE	96.0±3.2 / 97.7±2.7	344.15±53.13 / 337.36±80.56
	BA/BC	96.1±4.6 / 94.1±6.5	331.52±46.15 / 331.37±65.16
9 cpd	AE/ND	97.1±2.5 / 95.9±6.5	374.98±39.03 / 349.59±60.96
	FE/DE	97.0±2.6 / 94.2±5.3	349.10±52.58 / 350.80±60.68
	BA/BC	96.6±3.9 / 95.1±6.1	345.37±50.25 / 344.52±56.61

AE: Amblyopic Eye, ND: Non-dominant Eye, FE: Fellow Eye, DE: Dominant Eye, BA: Binoculus in Amblyopia, BC: Binoculus in Control

In anisometropic amblyopes and normal controls, a 3 (eye conditions: amblyopic eye, fellow eye and both eyes in amblyopia; non-dominant eye, dominant eye and both eyes in normal control group, respectively) × 3 (frequencies: 3, 6 and 9 cpd) univariate repeated measures ANOVA was conducted for ACC and RT. Compared with the corresponding eyes of two groups, a 2 (two groups: amblyopic eye and non-dominant eye, fellow eye and dominant eye, both eyes of amblyopia and both eyes of normal control)× 3 (frequencies: 3, 6 and 9 cpd) univariate repeated measures ANOVA was conducted for ACC and RT.

**Fig 2:**
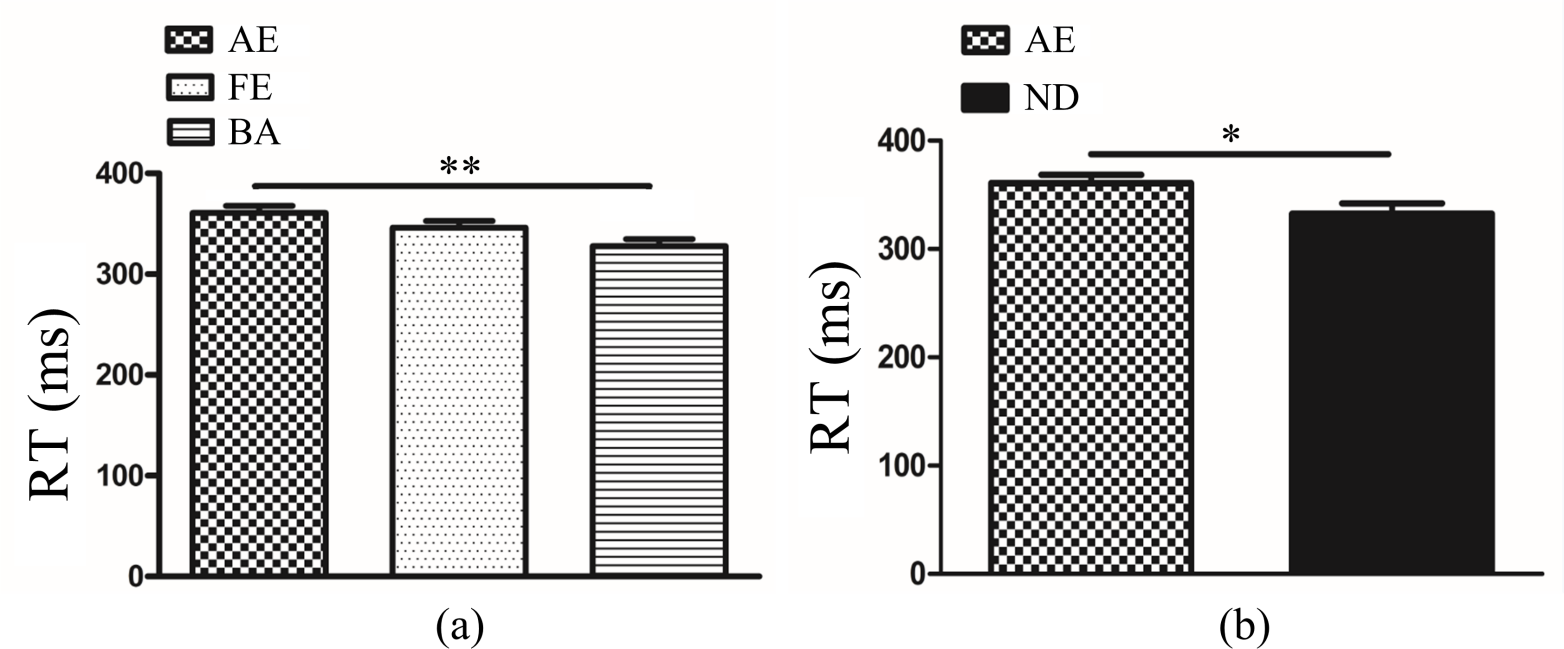
**(a)**. Reaction time (RT) in different eye conditions of amblypia (AE vs. BA *P<*0.01, AE vs. FE or FE vs. BA *P*>0.05). The Y-axis represents the response time range. Error bars indicate Standard Error (N_AA_ = 15; **P<0.01). **(b).** Reaction time (RT) between AE and DE. The Y-axis represents the response time range. Error bars indicate Standard Error (N_AA_ = 15 and N_NC_ = 10; *P<0.05). AE: Amblyopic Eye; FE: Fellow Eye, BA: Binoculus in Amblyopia; ND: Non-dominant Eye; N_AA_: Number of Anisometropic Amblyopia; N_NC_: Number of Normal Control.

In anisometropic amblyopes, ANOVA of ACC revealed that the main effects of eye and frequency were not significant (*F*_(2, 126)_ = 0.84, *P*>0.05; *F*_(2, 126)_ = 0.02, *P*>0.05). The interaction between eye condition and frequency was also not significant (*F*_(4, 126)_ = 0.63, *P*>0.05). The ANOVA of RT showed a significant main effect of eye condition (*F*_(2, 126)_ = 5.73, *P<*0.01, η^2^ = 0.08). Multiple comparison tests using the Tukey HSD method showed that RT was longer in the amblyopic eye (360.81±6.91 ms) when compared with both eyes (346.08±6.91 ms), but there was no significant difference between the fellow eye (327.78±6.91 ms) and amblyopic eye/both eyes (Fig 2A). The main effect of frequency was not significant (*F*_(2, 126)_ = 1.73, *P*>0.05), and the interaction between eye condition and frequency was also not significant (*F*_(4, 126)_ = 0.36, *P*>0.05).

In normal controls, ANOVA of ACC revealed that the main effects of eye and frequency were not significant (*F*_(2, 81)_ = 0.18, *P*>0.05; *F*_(2, 81)_ = 0.47, *P*>0.05, respectively). The interaction between eye condition and frequency was also not significant (*F*_(4, 81)_ = 0.56, *P*>0.05). The ANOVA of RT showed that the main effects of eye and frequency were not significant (*F*_(2, 81)_ = 0.01, *P*>0.05; *F*_(2, 81)_ = 1.43, *P*>0.05, respectively). The interaction between eye condition and frequency was also not significant (*F*_(4, 81)_ = 0.07, *P*>0.05).

The ANOVA of ACC of amblyopic eye and non-dominant eye revealed that the main effects of group and frequency were not significant (*F*_(1,69)_ = 1.83, *P*>0.05; *F*_(2,69)_ = 0.04, *P*>0.05). The interaction between group and frequency was also not significant (*F*_(2,69)_ = 0.47, *P*>0.05). The ANOVA of RT showed a significant main effect of group (*F*_(1,69)_ = 5.35, *P<*0.05, η^2^ = 0.07).Multiple comparison tests using the Tukey HSD method showed that RT was longer in the amblyopic eye (360.81±7.72 ms) than non-dominant eye (332.57±9.46 ms) (Fig 2B). The main effect of frequency was not significant (*F*_(2, 69)_ = 1.90, *P*>0.05) and the interaction between group and frequency was also not significant (*F*_(2, 69)_ = 0.17, *P*>0.05).

The ANOVA of ACC of fellow eye and dominant eye revealed that the main effects of group and frequency were not significant (*F*_(1, 69)_ = 0.43, *P*>0.05; *F*_(2, 69)_ = 0.86, *P*>0.05). The interaction between group and frequency was also not significant (*F*_(2, 69)_ = 2.15, *P*>0.05). The ANOVA of RT showed that the main effects of group and frequency were not significant (*F*_(1, 69)_ = 0.77, *P*>0.05; *F*_(2,69)_ = 0.74, *P*>0.05). The interaction between group and frequency was also not significant (*F*_(2, 69)_ = 0.52, *P*>0.05).

The ANOVA of ACC in binoculus between the amblyopia and normal control revealed that the main effects of group and frequency were not significant (*F*_(1,69)_ = 0.60, *P*>0.05; *F*_(2,69)_ = 0.33, *P*>0.05). The interaction between group and frequency was also not significant (*F*_(2,69)_ = 0.45, *P*>0.05). The ANOVA of RT showed that the main effects of group and frequency were not significant (*F*_(1,69)_ = 0.08, *P*>0.05; *F*_(2,69)_ = 0.93, *P*>0.05). The interaction between group and frequency was also not significant (*F*_(2,69)_ = 0.04, *P*>0.05).

### ERP analysis

**Fig 3:**
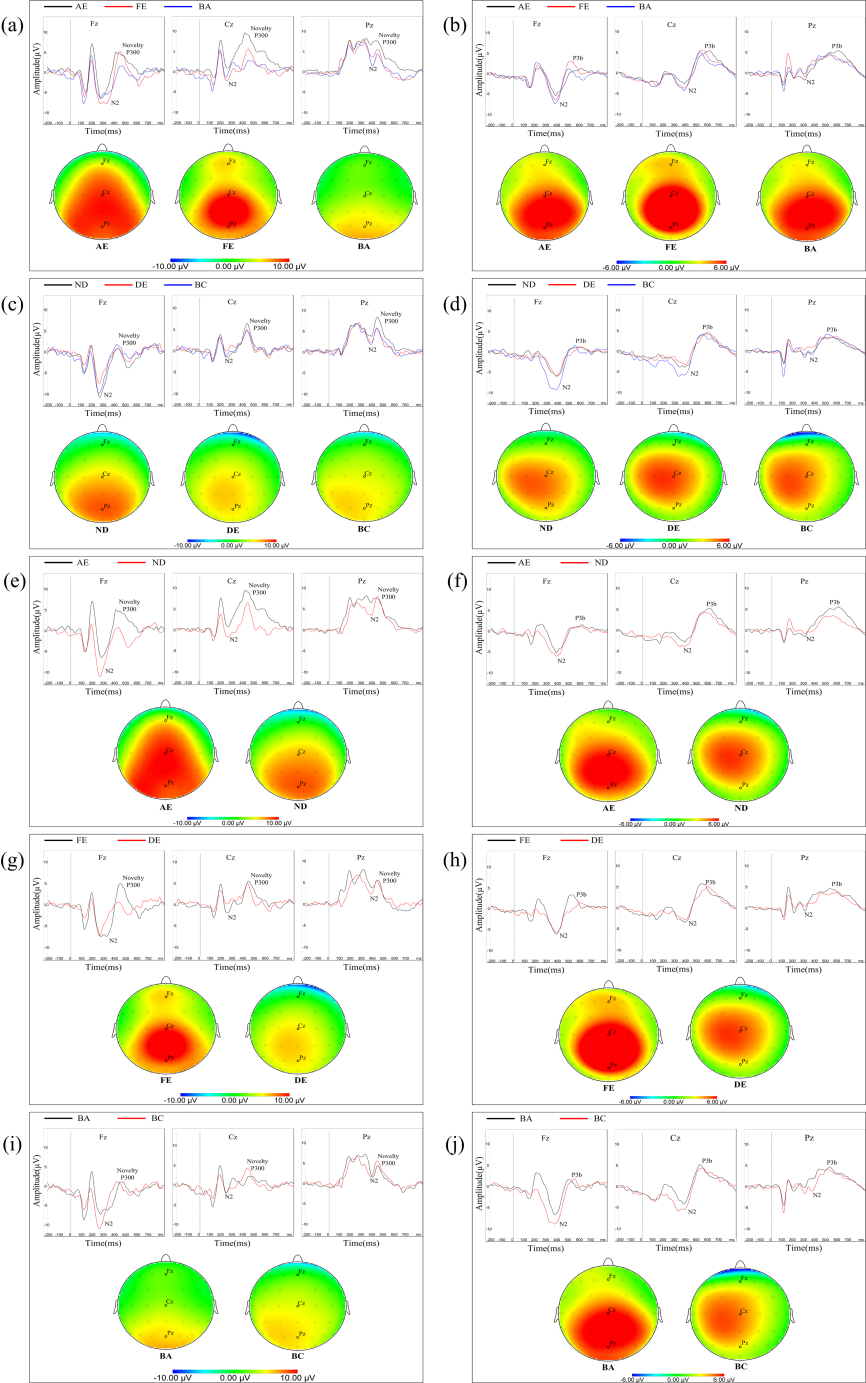
(**a**). Upper panel: Grand average ERP waveforms were elicited by novelty stimuli over Fz, Cz and Pz electrode in anisometropic amblyopes respectively. Below panel: Topographic amplitude maps for each novelty P300 from AE, FE and BE respectively (AE: black solid line; FE: red solid line; BA: blue solid line). (**b**). Upper panel: Grand average ERP waveforms was elicited by target stimuli over Fz, Cz and Pz electrode in anisometropic amblyopes respectively. Below panel: Topographic amplitude maps for each P3b from AE, FE and BE respectively. (**c**). Upper panel: Grand average ERP waveforms were elicited by novelty stimuli over Fz, Cz and Pz electrode in normal controls respectively. Below panel: Topographic amplitude maps for each novelty P300 from ND, DE and BC respectively (ND: black solid line; DE: red solid line; BC: blue solid line). (**d**). Upper panel: Grand average ERP waveforms were elicited by target stimuli over Fz, Cz and Pz electrode in normal control respectively. Below panel: Topographic amplitude maps for each P3b from ND, DE and BC respectively. (**e**). Upper panel: Grand average ERP waveforms were elicited by novelty stimuli over Fz, Cz and Pz electrode from AE and ND respectively. Below panel: Topographic amplitude maps for each novelty P300 from AE and ND respectively (AE: black solid line; ND: red solid line). (**f**). Upper panel: Grand average ERP waveforms was elicited by target stimuli over Fz, Cz and Pz electrode from AE and ND respectively. Below panel: Topographic amplitude maps for each P3b from AE and ND respectively. (**g**). Upper panel: Grand average ERP waveforms were elicited by novelty stimuli over Fz, Cz and Pz electrode from FE and DE respectively. Below panel: Topographic amplitude maps for each novelty P300 from FE and DE respectively (FE: black solid line; DE: red solid line). (**h**). Upper panel: Grand average ERP waveforms were elicited by target stimuli over Fz, Cz and Pz electrode from FE and DE respectively. Below panel: Topographic amplitude maps for each P3b from FE and DE respectively. (**i**). Upper panel: Grand average ERP waveforms were elicited by novelty stimuli over Fz, Cz and Pz electrode from BA and BC respectively. Below panel: Topographic amplitude maps for each novelty P300 from BA and BC respectively (BA: black solid line; BC: red solid line). (**j**). Upper panel: Grand average ERP waveforms were elicited by target stimuli over Fz, Cz and Pz electrode from BA and BC respectively. Below panel: Topographic amplitude maps for each P3b from BA and BC respectively. N_AA_: Number of Anisometropic Amblyopia; N_NC_: Number of Normal Control; AE: Amblyopic Eye; FE: Fellow eye; BA: Binoculus in Amblyopia; ND: Non-dominant Eye; DE: Dominant Eye; BC: Binoculus in Control; F: Frontal Electrode; C: Central Electrode; P: Parietal Electrode. The X-axis represents time, where 0 indicates the onset of the target stimulus. The Y-axis represents the wave amplitude in grand average ERP waveforms. The dots represent scalp electrode positions. Contours connect points of equal amplitude on the waves in the topographic distribution.

The latency of N2 (200–400 ms) and the latency and amplitude of novelty P300 (250–550 ms) and P3b (300–600 ms) were analyzed over the frontal, central and parietal midline electrodes (F_Z_, C_Z_, P_Z_). The grand average ERP waveforms and P300 topographic distributions of novelty and target stimuli from the amblyopes and normal controls in the different eye conditions are represented in Fig 3A and 3B and Fig 3C and 3D, respectively. The grand average ERP waveforms and P300 topographic distributions of novelty and target stimuli from the amblyopic eyes and non-dominant eyes, fellow eyes and dominant eyes and binoculus of two groups were represented in Fig 3E and 3F, Fig 3G and 3H and Fig 3I and 3J, respectively.

#### N2 latency for novel stimuli

In anisometropic amblyopia group and normal control group, we conducted a one-way ANOVA (eye conditions: amblyopic eye, fellow eye and both eyes in amblyopia group; non-dominant eye, dominant eye and both eyes in normal control group, respectively) of N2 latency for novel stimuli. The results showed that N2 latency was not significantly different among the three different viewing conditions in each group (*F*_(2,396)_ = 1.21, *P*>0.05; *F*_(2,261)_ = 1.94, *P*>0.05).When compared with the corresponding eyes of two groups (amblyopic eye and non-dominant eye, fellow eye and dominant eye, binoculus in amblyopia and binoculus in normal control), we conducted independent sample t test of N2 latency for novel stimuli. The results showed that N2 latency was not significantly different between the corresponding eyes of two groups (*t*_219_ = 0.26, *P*>0.05; *t*_219_ = 0.04, *P*>0.05; *t*_219_ = 0.28, *P*>0.05).

#### Novelty P300 latency and amplitude

**Fig 4:**
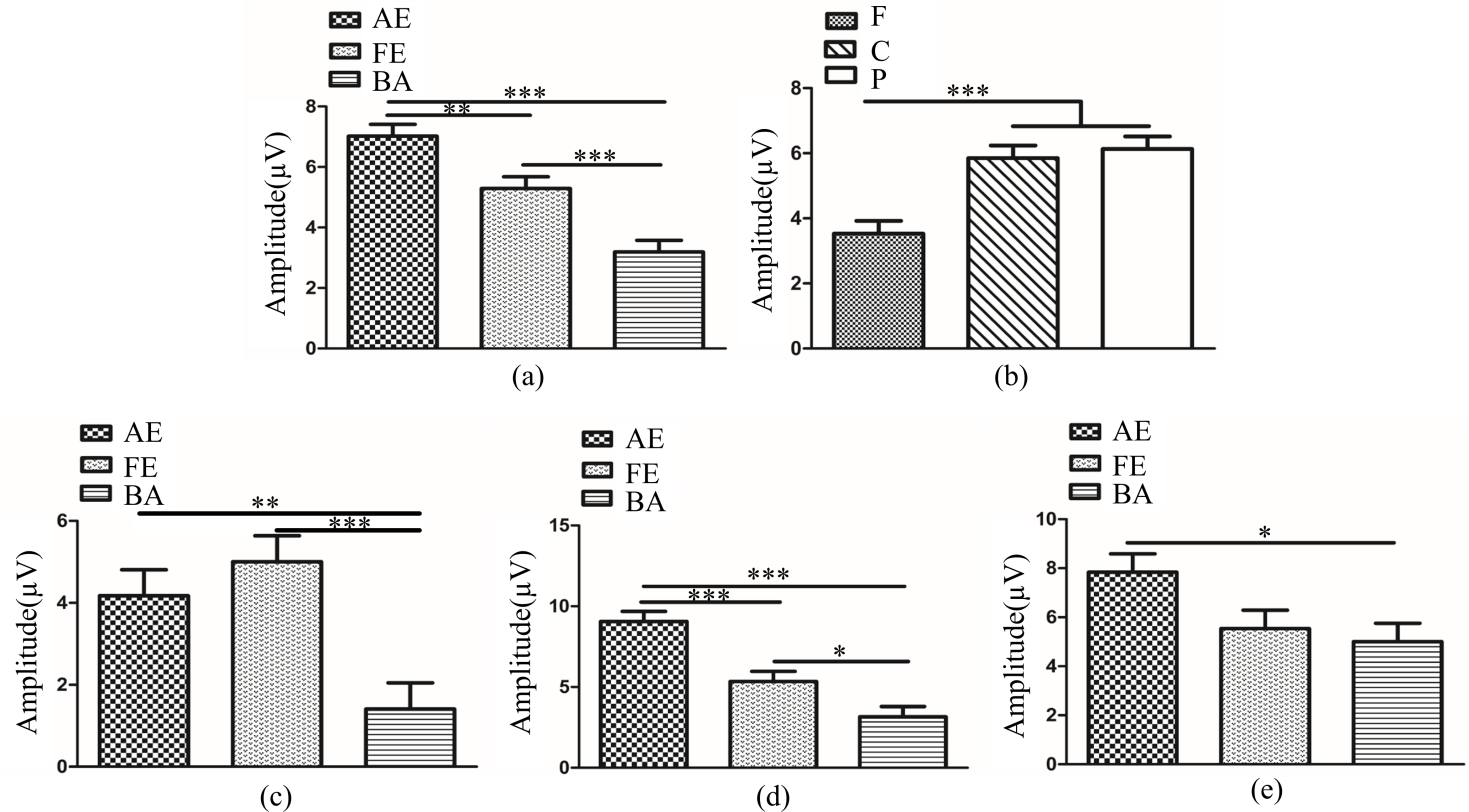
(**a**). Novelty P300 amplitude in different eye conditions in amblyopia (AE vs. FE *P<*0.01; AE vs. BA *P<*0.001; FE vs. BA *P<*0.001). (**b**). Novelty P300 amplitude in different brain regions in amblyopia (F vs. C *P*<0.001; F vs. P *P<*0.001; C vs. P *P*>0.05). (**c**). Novelty P300 amplitude in different eye conditions of amblyopia over Fz electrode (AE vs. FE *P>*0.05; AE vs. BA *P<*0.01; FE vs. BA *P<*0.001). (**d**). Novelty P300 amplitude in different eye conditions of amblyopia over Cz electrode (AE vs. FE *P<*0.001; AE vs. BA *P<*0.001; FE vs. BA *P<*0.05). (**e**). Novelty P300 amplitude in different eye conditions of amblyopia over Pz electrode (AE vs. FE *P>*0.05; AE vs. BA *P<*0.05; FE vs. BA *P>*0.05). The Y-axis represents the wave amplitude range. Error bars indicate Standard Error (N_AA_ = 15; *P<0.05; **P<0.01; ***P<0.001). AE: Amblyopic Eye; FE: Fellow Eye; BA: Binoculus in Amblyopia; F: Frontal Lobe, C: Central Lobe, P: Parietal Lobe; N_AA_: Number of Anisometopic Amblyopia.

In anisometropia group, novelty P300 latency and amplitude were subjected to a 3 (eye conditions: amblyopic eye, fellow eye and both eyes) × 3 (brain regions: frontal, central and parietal lobe) ANOVA for novel stimuli. The analysis of novelty P300 latency indicated that the main effects of eye condition and brain region were not significant (*F*_(2,396)_ = 1.92, *P*>0.05; *F*_(2,396)_ = 1.10, *P*>0.05). The interaction between eye condition and brain region was also not significant (*F*_(4,396)_ = 0.28, *P*>0.05). Analysis of the novelty P300 amplitude showed a significant main effect of eye condition (*F*_(2,396)_ = 24.13., *P*<0.001, η^2^ = 0.11). Multiple comparison tests using the Tukey HSD method showed that the amplitude was highest in the amblyopic eye (7.02±0.39 μV), followed by the fellow eye (5.29±0.39 μV), and was smallest in both eyes (3.19±0.39 μV) (Fig 4A). The main effect of brain region was significant (*F*_(2,396)_ = 13.35, *P<*0.001, η^2^ = 0.06). Multiple comparison tests using the Tukey HSD method showed that the amplitude over the central (5.85±0.39 μV) and parietal lobes (6.13±0.39 μV) was larger than the frontal area (3.53±0.39 μV) (Fig 4B). The interaction between eye condition and brain region was significant (*F*_(4,396)_ = 3.97, *P<*0.01, η^2^ = 0.04). Simple-effects analysis showed that the difference in amplitude among the three eye conditions was statistically significant over the frontal, central and parietal lobes. The novelty P300 amplitude in the amblyopic eye (4.17±0.64 μV) and fellow eye (5.00±0.64 μV) were larger than in the binocular condition (1.41±0.64 μV) over the frontal lobe (Fig 4C). Over the central lobe, the novelty P300 amplitude in the amblyopic eye (9.05±0.63 μV) was largest, followed by the fellow eye (5.34±0.63 μV), and was smallest in the binocular condition (3.16±0.63 μV) (Fig 4D). Over the parietal region, the amplitude of novelty P300 was larger in the amblyopic eye (7.84±0.75 μV) compared with both eyes (5.00±0.75 μV) (Fig 4E). There was no significant difference between fellow eye (5.54±0.75 μV) and amblyopic eye/both eyes.

**Fig 5:**
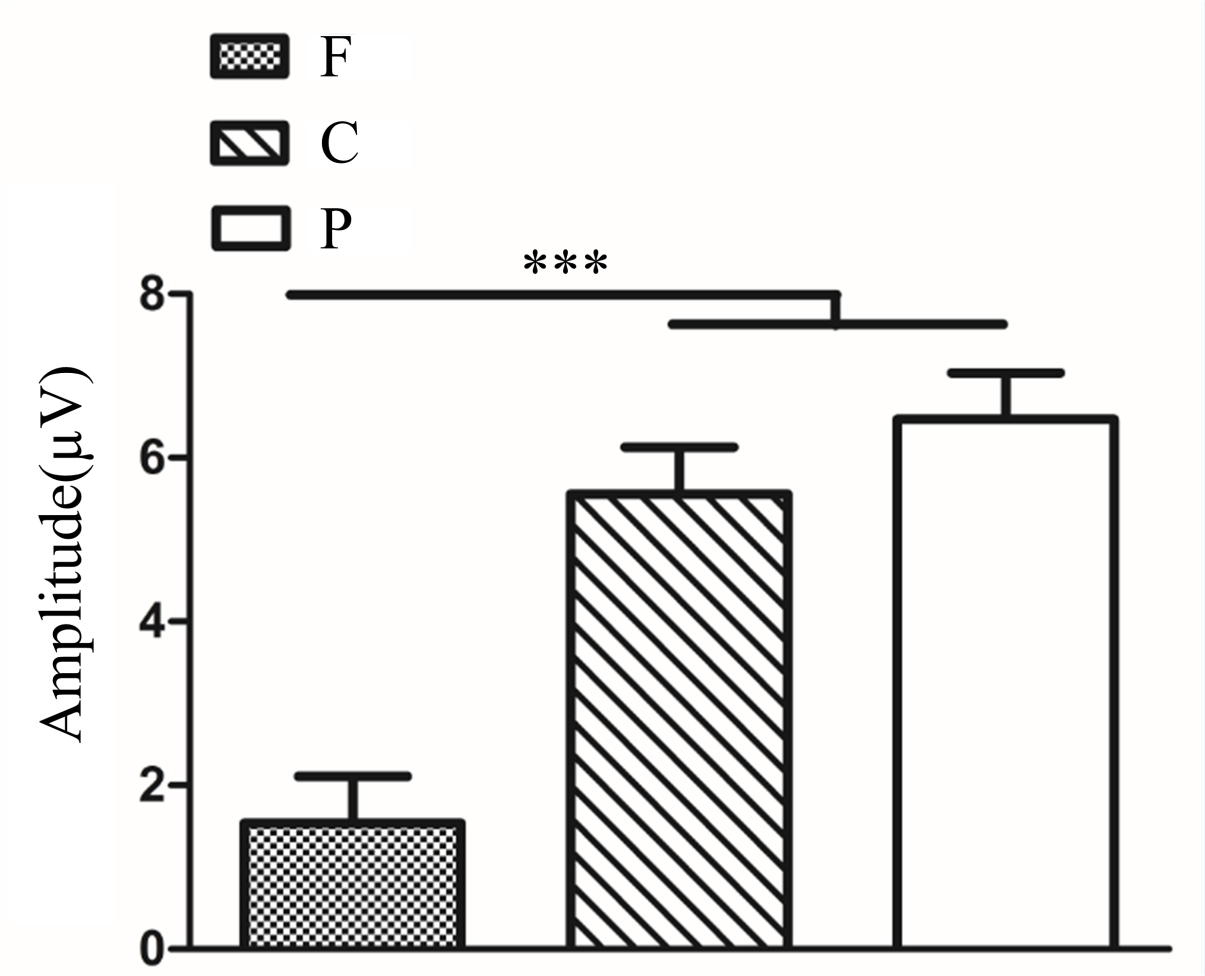
Novelty P300 amplitude in different brain regions in normal control (F vs. C *P*<0.001; F vs. P *P<*0.001; C vs. P *P*>0.05). The Y-axis represents the wave amplitude range. Error bars indicate Standard Error (N_NC_ = 10; ***P<0.001). F: Frontal Lobe; C: Central Lobe; P: Parietal Lobe; N_NC_: Number of Normal Control.

In normal control group, novelty P300 latency and amplitude were subjected to a 3 (eye conditions: non-dominant eye, dominant eye and both eyes) × 3 (brain regions: frontal, central and parietal lobe) ANOVA for novel stimuli. The analysis of novelty P300 latency indicated that the main effects of eye condition and brain region were not significant (*F*_(2,261)_ = 0.21, *P*>0.05; *F*_(2,261)_ = 0.09, *P*>0.05). The interaction between eye condition and brain region was also not significant (*F*_(4,261)_ = 0.04, *P*>0.05). Analysis of the novelty P300 amplitude showed a significant main effect of brain region was significant (*F*_(2,261)_ = 20.90, *P<*0.001, η^2^ = 0.14). Multiple comparison tests using the Tukey HSD method showed that the amplitude over the central (5.56±0.57 μV) and parietal lobes (6.47±0.57 μv) was larger than the frontal area (1.54±0.57 μV) (Fig 5). The main effect of eye condition and the interaction between eye condition and brain region were not significant (*F*_(2,261)_ = 2.45, *P*>0.05; *F*_(4,261)_ = 1.10, *P*>0.05).

**Fig 6:**
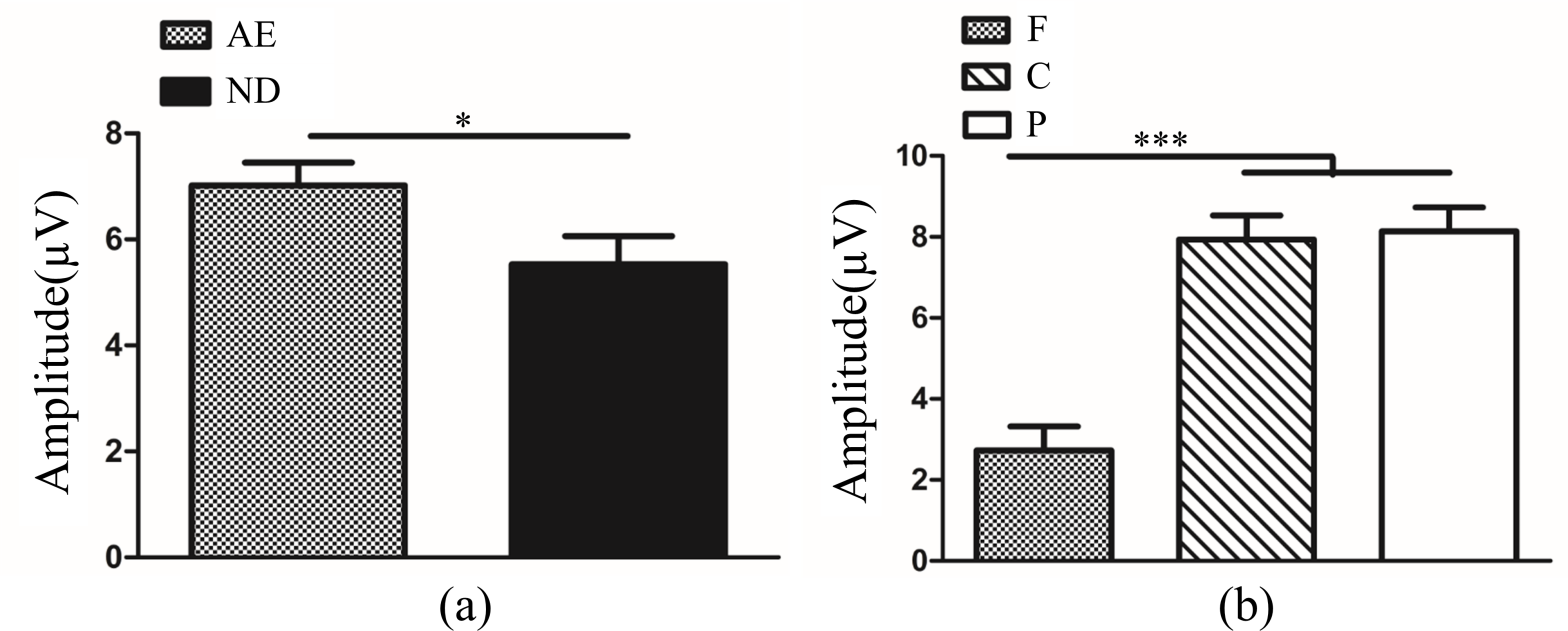
(**a**). Novelty P300 amplitude between AE and ND (*P*<0.05). (**b**). Novelty P300 amplitude in different brain regions in AE and ND (F vs. C *P*<0.001; F vs. P *P<*0.001; C vs. P *P*>0.05). The Y-axis represents the wave amplitude range. Error bars indicate Standard Error (N_AA_ = 15 and N_NC_ = 10; * *P*<0.05; ***P<0.001). AE: Amblyopic Eye; ND: Non-dominant Eye; F: Frontal Lobe; C: Central Lobe; P: Parietal Lobe; N_AA_: Number of Anisometopic Amblyopia; N_NC_: Number of Normal Control.

In amblyopic eye and non-dominant eye, novelty P300 latency and amplitude were subjected to a 2 (two groups: amblyopic eye and non-dominant eye) × 3 (brain regions: frontal, central and parietal lobe) ANOVA for novel stimuli. The analysis of novelty P300 latency indicated that the main effects of group and brain region were not significant (*F*_(1,219)_ = 0.08, *P*>0.05; *F*_(2,219)_ = 0.01, *P*>0.05). The interaction between group and brain region was also not significant (*F*_(2,219)_ = 0.02, *P*>0.05). Analysis of the novelty P300 amplitude showed a significant main effect of group (*F*_(1,219)_ = 4.77, *P<*0.05, η^2^ = 0.02). Multiple comparison tests using the Tukey HSD method showed that the amplitude was higher in the amblyopic eye (7.02±0.43 μV) than non-dominant eye (5.53±0.53 μV) (Fig 6A). Analysis of the novelty P300 amplitude showed a significant main effect of brain region was significant (*F*_(2,219)_ = 26.87, *P<*0.001, η^2^ = 0.20). Multiple comparison tests using the Tukey HSD method showed that the amplitude over the central (7.94±0.59 μV) and parietal lobes (8.15±0.59 μv) was larger than the frontal area (2.73±0.59 μV) (Fig 6B). The interaction between group and brain region was not significant (*F*_(2,219)_ = 2.46, *P*>0.05).

**Fig 7:**
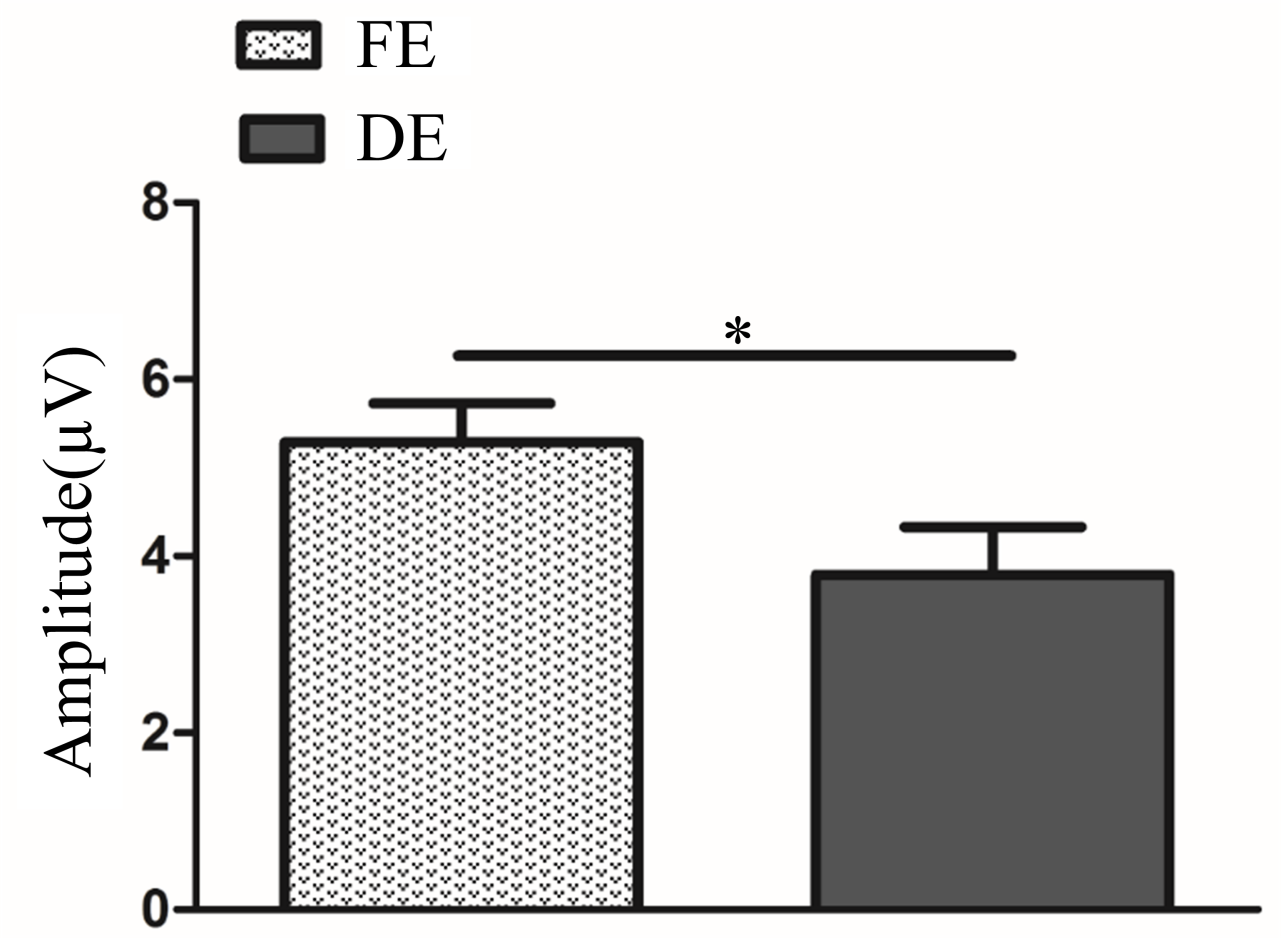
Novelty P300 amplitude between FE and DE (*P*<0.05). The Y-axis represents the wave amplitude range. Error bars indicate Standard Error (N_AA_ = 15 and N_NC_ = 10; * *P*<0.05). FE: Fellow Eye; DE: Dominant Eye; N_AA_: Number of Anisometopic Amblyopia; N_NC_: Number of normal control.

In fellow eye and dominant eye, novelty P300 latency and amplitude were subjected to a 2 (two groups: fellow eye and dominant eye) × 3 (brain regions: frontal, central and parietal lobe) ANOVA for novel stimuli. The analysis of novelty P300 latency indicated that the main effects of group and brain region were not significant (F_(1,219)_ = 3.45, *P*>0.05; F_(2,219)_ = 0.12, *P*>0.05). The interaction between group and brain region was also not significant (F_(2,219)_ = 0.31, *P*>0.05). Analysis of the novelty P300 amplitude showed a significant main effect of group (F_(1,219)_ = 4.63, *P*<0.05, η2 = 0.02). Multiple comparison tests using the Tukey HSD method showed that the amplitude was higher in the fellow eye (5.29±0.44 μV) than dominant eye (3.79±0.54 μV) (Fig 7). Analysis of the novelty P300 amplitude showed a significant main effect of brain region was significant (F_(2,219)_ = 3.95, *P*<0.05, η2 = 0.04). However, multiple comparison tests using the Tukey HSD method showed that the amplitude was no significant difference between any two lobes. The interaction between group and brain region was not significant (*F*_(2,219)_ = 2.46, *P*>0.05).

**Fig 8:**
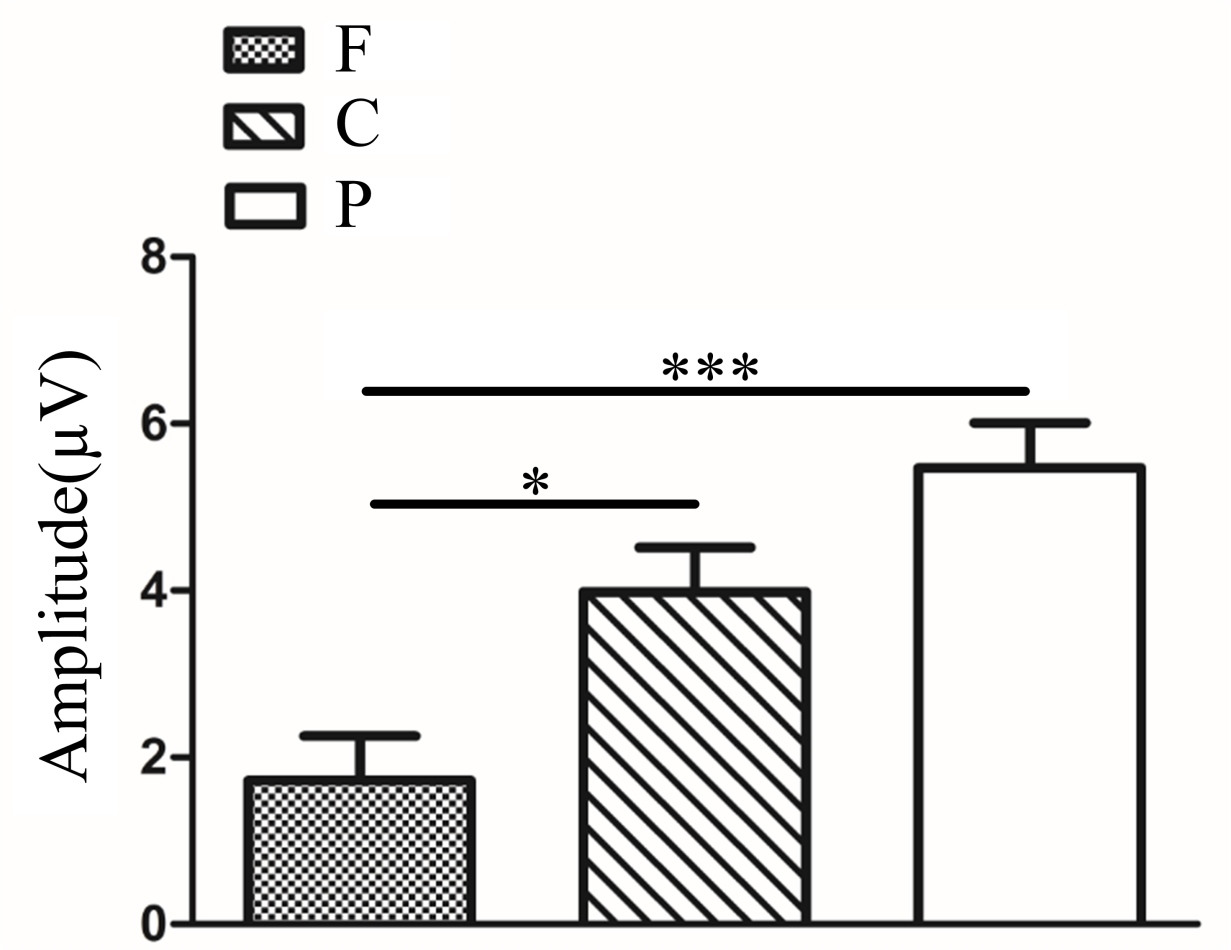
Novelty P300 amplitude in different brain regions in BA and BC (F vs. C *P*<0.05; F vs. P *P*<0.001; C vs. P *P*>0.05). The Y-axis represents the wave amplitude range. Error bars indicate Standard Error (N_AA_ = 15 and N_NC_ = 10; * *P*<0.05; ***P<0.001). BA: Binoculus in Amblyopia; BC: Binoculus in Normal Control; F: Frontal Lobe; C: Central Lobe; P: Parietal Lobe; N_AA_: Number of Anisometopic Amblyopia; N_NC_: Number of normal control.

In binoculus in amblyopia and binoculus in normal control, novelty P300 latency and amplitude were subjected to a 2 (two groups: binoculus in amblyopia and binoculus in normal control) × 3 (brain regions: frontal, central and parietal lobe) ANOVA for novel stimuli. The analysis of novelty P300 latency indicated that the main effects of group and brain region were not significant (*F*_(1,219)_ = 1.75, *P*>0.05; *F*_(2,219)_ = 0.05, *P*>0.05). The interaction between group and brain region was also not significant (*F*_(2,219)_ = 0.81, *P*>0.05). Analysis of the novelty P300 amplitude showed a significant main effect of brain region was significant (*F*_(2,219)_ = 12.28, *P<*0.001, η^2^ = 0.10). Multiple comparison tests using the Tukey HSD method showed that the amplitude over the central (3.98±0.54 μV) and parietal lobes (5.47±0.54 μv) was larger than the frontal area (1.72±0.54 μV) (Fig 8). The main effect of group and the interaction between group and brain region was not significant (*F*_(1,219)_ = 2.94, *P*>0.05; *F*_(2,219)_ = 2.46, *P*>0.05).

#### N2 latency for target stimuli

Moreover, we conducted a one-way ANOVA (eye conditions: amblyopic eye, fellow eye and both eyes in amblyopia group; non-dominant eye, dominant eye and both eyes in normal control group, respectively) of N2 latency for target stimuli in amblyopes. The results showed that N2 latency was not significantly different between the three different viewing conditions in each group (*F*(_2,378)_ = 1.75, *P*>0.05; *F*(_2,243)_ = 0.24, *P*>0.05). When compared with the corresponding eyes of two groups (amblyopic eye and non-dominant eye, fellow eye and dominant eye, binoculus in amblyopia and binoculus in normal control), we conducted independent sample t test of N2 latency for target stimuli. The results showed that N2 latency was not significantly different between the corresponding eyes of two groups (*t*_207_ = 0.06, *P*>0.05; *t*_207_ = 1.06, *P*>0.05; *t*_219_ = 0.30, *P*>0.05y).

#### P3b latency and amplitude

**Fig 9:**
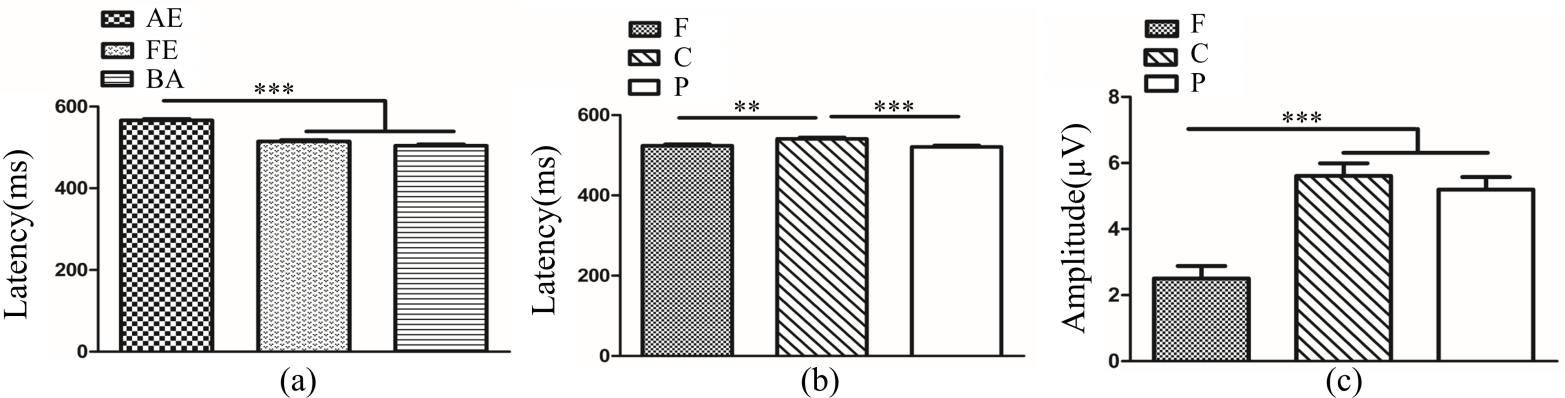
(**a**). P3b latency in different eye conditions in anisometropic amblyopia (AE vs. FE *P<*0.001; AE vs. BA *P <*0.001; FE vs. BA *P*>0.05). (**b**). P3b latency in different brain regions in anisometropic amblyopia (F vs. C *P*<0.01; F vs. P *P*>0.05; C vs. P *P<*0.001). (**c**). P3b amplitude in different brain regions in anisometropic amblyopia (F vs. C *P*<0.001; F vs. P *P<*0.001; C vs. P *P*>0.05). The Y-axis represents the wave latency or amplitude range. Error bars indicate Standard Error (N_AA_ = 15; **P<0.01; ***P<0.001). AE: Amblyopic Eye; FE: Fellow Eye; BA: Binoculus in Amblyopia; F: Frontal Lobe; C: Central Lobe; P: Parietal Lobe; N_AA:_ Number of Anisometropic Amblyopia.

In amblyopic group, we conducted a 3 (eye conditions: amblyopic eye, fellow eye and both eyes) × 3 (frequencies: 3, 6 and 9 cpd) × 3 (brain regions: frontal, central and parietal lobe) ANOVA of latency and amplitude for P3b in target stimuli. Analysis of P3b latency showed a significant main effect of eye condition (*F*_(2,378)_ = 89.85, *P*<0.001, η^2^ = 0.32). Multiple comparison tests using the Tukey HSD method showed that the latency in the amblyopic eye (566.33±3.51 ms) was longer than that in the fellow eye (514.79±3.51 ms) and both eyes (504.00±3.51 ms), and there was no difference between the fellow eye and both eyes (Fig 9A). The main effect of frequency was not significant (*F*_(2,378)_ = 0.67, *P*>0.05), while the main effect of brain region was significant (*F*_(2,378)_ = 9.37, *P<*0.001, η^2^ = 0.05). Multiple comparison tests using the Tukey HSD method showed that a longer latency occurred over the central lobe (540.68±3.51 ms) compared with that over the frontal lobe (523.65±3.51 ms) and parietal lobe (520.79±3.51 ms) (Fig 9B). The interactions between eye condition and frequency, between eye condition and brain region, and between frequency and brain region were not significant (*F*_(4,378)_ = 0.47, *P*>0.05; *F*_(4,378)_ = 2.31, *P*>0.05; *F*_(4,378)_ = 0.29, *P*>0.05). The interaction among eye condition, frequency and brain region was also not significant (*F*_(8,378)_ = 0.50, *P*>0.05). P3b amplitude analysis showed a significant main effect of brain region (*F*_(2,378)_ = 20.30, *P*<0.001, η^2^ = 0.10). Multiple comparison tests using the Tukey HSD method showed that the amplitude over the central (5.61±0.38 μV) and parietal regions (5.20±0.38 μV) was higher than in the frontal lobe (2.50±0.38 μV) (Fig 9C). The main effects of eye condition and frequency were not significant (*F*_(2,378)_ = 2.48, *P*>0.05; *F*_(2,378)_ = 0.28, *P*>0.05). The interactions between eye condition and frequency, between eye condition and brain region, and between frequency and brain region were also not significant (*F*_(4,378)_ = 0.76, *P*>0.05; *F*_(4,378)_ = 1.03, *P*>0.05; *F*_(4,378)_ = 0.65, *P*>0.05). The interaction among eye condition, frequency and brain region was not significant (*F*_(8,378)_ = 0.15, *P*>0.05).

**Fig 10:**
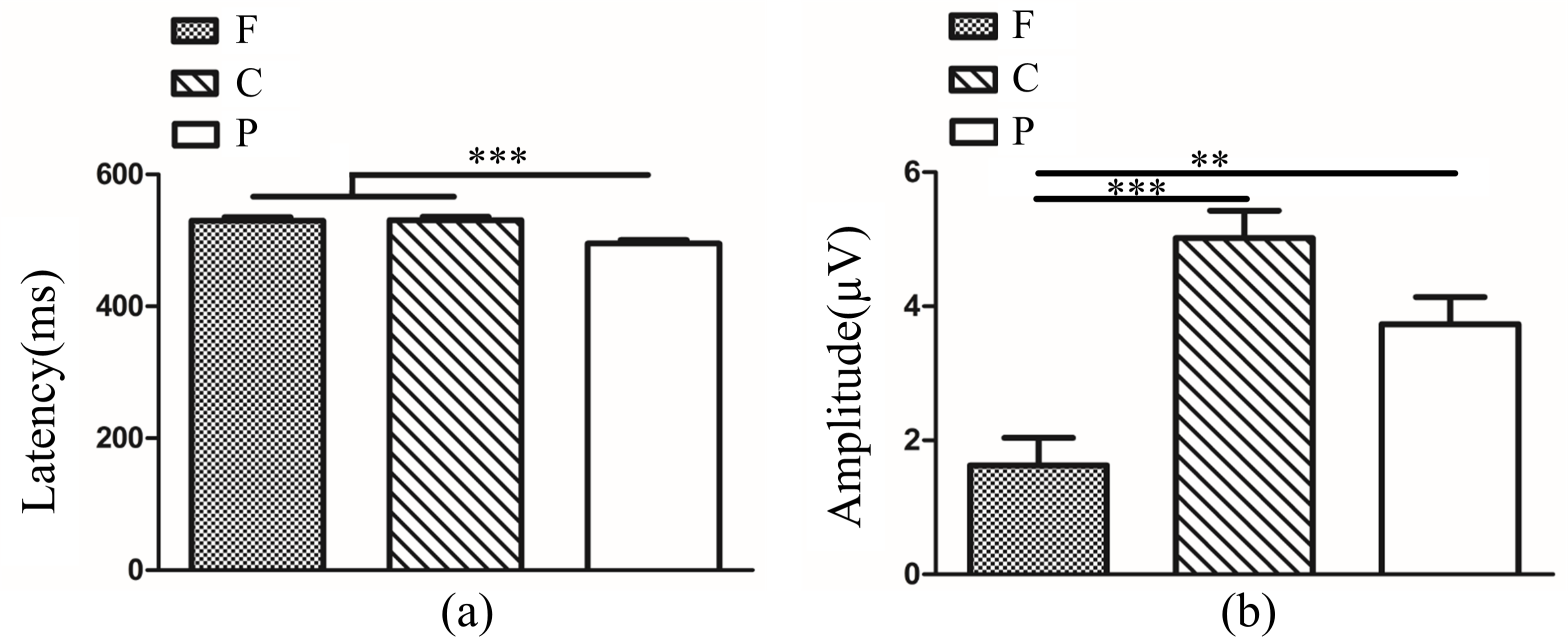
**(a)**. P3b latency in different brain regions in normal control (F vs. C *P>*0.05; F vs. P *P<*0.001; C vs. P *P<*0.001). **(b).** P3b amplitude in different brain regions in normal control (F vs. C *P*<0.001; F vs. P *P<*0.01; C vs. P *P*>0.05). The Y-axis represents the wave latency or amplitude range. Error bars indicate Standard Error (N_NC_ = 10; **P<0.01; ***P<0.001). F: Frontal Lobe; C: Central Lobe; P: Parietal Lobe; N_NC_: Number of Normal Control.

In normal control group, we conducted a 3 (eye conditions: non-dominant eye, dominant eye and both eyes) × 3 (frequencies: 3, 6 and 9 cpd) × 3 (brain regions: frontal, central and parietal lobe) ANOVA of latency and amplitude for P3b in target stimuli. Analysis of P3b latency showed a significant main effect of brain region (*F*_(2,243)_ = 14.34, *P<*0.001, η^2^ = 0.11). Multiple comparison tests using the Tukey HSD method showed that a longer latency occurred over the frontal lobe (529.79±5.32 ms) and central lobe (530.52±5.32 ms) compared with that over the parietal lobe (495.26±5.32 ms) (Fig 10A). The main effects of eye condition and frequency were not significant (*F*_(2,243)_ = 2.70, *P*>0.05; *F*_(2,378)_ = 0.34, *P*>0.05). The interactions between eye condition and frequency, between eye condition and brain region, and between frequency and brain region were not significant (*F*_(4,243)_ = 1.48, *P*>0.05; *F*_(4,243)_ = 0.48, *P*>0.05; *F*_(4,243)_ = 0.17, *P*>0.05). The interaction among eye condition, frequency and brain region was also not significant (*F*_(8,243)_ = 0.43, *P*>0.05). P3b amplitude analysis showed a significant main effect of brain region (*F*_(2,243)_ = 17.11, *P*<0.001, η^2^ = 0.12). Multiple comparison tests using the Tukey HSD method showed that the amplitude over the central (5.02±0.41 μV) and parietal regions (3.73±0.41 μV) was higher than in the frontal lobe (1.63±0.41 μV) (Fig 10B). The main effects of eye condition and frequency were not significant (*F*_(2,243)_ = 0.38, *P*>0.05; *F*_(2,243)_ = 2.08, *P*>0.05). The interactions between eye condition and frequency, between eye condition and brain region, and between frequency and brain region were also not significant (*F*_(4,243)_ = 0.58, *P*>0.05; *F*_(4,243)_ = 0.29, *P*>0.05; *F*_(4,243)_ = 0.24, *P*>0.05). The interaction among eye condition, frequency and brain region was not significant (*F*_(8,243)_ = 0.37, *P*>0.05).

**Fig 11:**
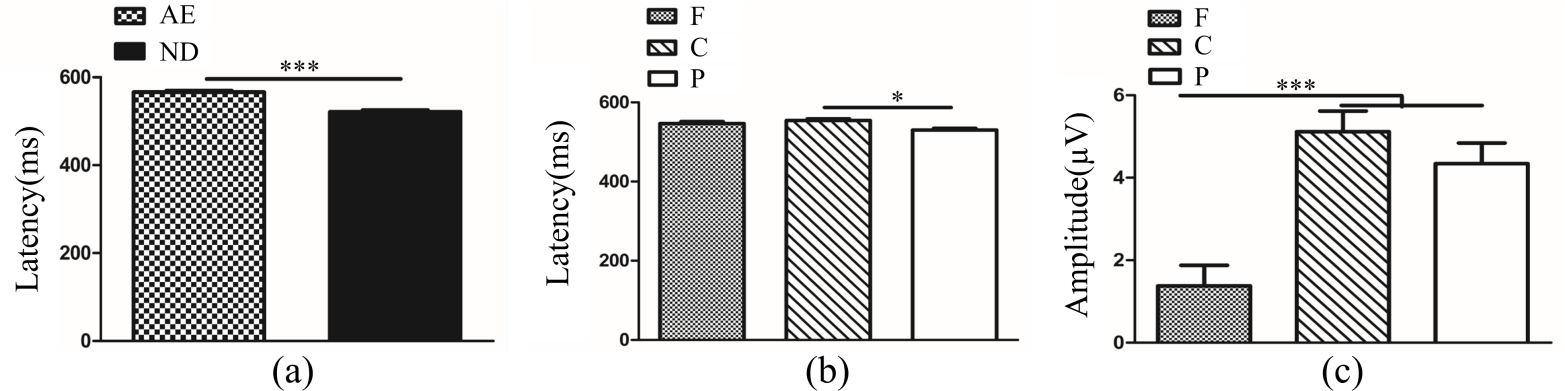
(**a**). P3b latency between AE and ND (*P*<0.001). (**b**). P3b latency in different brain regions in AE and ND (F vs. C *P*>0.05; F vs. P *P*>0.05; C vs. P *P<*0.05). (**c**). P3b amplitude in different brain regions in AE and ND (F vs. C *P*<0.001; F vs. P *P<*0.001; C vs. P *P*>0.05). The Y-axis represents the wave latency or amplitude range. Error bars indicate Standard Error (N_AA_ = 15 and N_NC_ = 10, * *P*<0.05; ***P<0.001). AE: Amblyopic Eye; ND: Non-dominant Eye; F: Frontal Lobe; C: Central Lobe; P: Parietal Lobe; N_AA_: Number of Anisometopic Amblyopia; N_NC_: Number of Normal Control.

In amblyopic eye and non-dominant eye, we conducted a 2 (two groups: amblyopic eye and non-dominant eye) × 3 (frequencies: 3, 6 and 9 cpd) × 3 (brain regions: frontal, central and parietal lobe) ANOVA of latency and amplitude for P3b in target stimuli. Analysis of P3b latency showed a significant main effect of group (*F*_(1,207)_ = 69.92, *P<*0.001, η^2^ = 0.25). Multiple comparison tests using the Tukey HSD method showed that the latency in the amblyopic eye (566.33±3.42 ms) was longer than non-dominant eye (521.09±4.19 ms) (Fig 11A). There was a significant main effect of brain region (*F*_(2,207)_ = 6.85, *P<*0.01, η^2^ = 0.06). Multiple comparison tests using the Tukey HSD method showed that a longer latency occurred over the central lobe (554.03±4.69 ms) compared with that over the parietal lobe (530.15±4.69 ms) and there was no difference between the frontal lobe (546.94±4.69 ms) and central/parietal lobe (Fig 11B). The main effect of frequency was not significant (*F*_(2,207)_ = 2.25, *P*>0.05). The interactions between group and frequency, between group and brain region, and between frequency and brain region were not significant (*F*_(2,207)_ = 1.57, *P*>0.05; *F*_(2,207)_ = 0.67, *P*>0.05; *F*_(4,207)_ = 0.19, *P*>0.05). The interaction among group, frequency and brain region was also not significant (*F*_(4,207)_ = 0.80, *P*>0.05). P3b amplitude analysis showed a significant main effect of brain region (*F*_(2,207)_ = 15.87, *P*<0.001, η^2^ = 0.13). Multiple comparison tests using the Tukey HSD method showed that the amplitude over the central (5.12±0.50 μV) and parietal regions (4.33±0.50 μV) was higher than in the frontal lobe (1.38±0.50 μV) (Fig 11C). The main effects of group and frequency were not significant (*F*_(1,207)_ = 2.45, *P*>0.05; *F*_(2,207)_ = 0.88, *P*>0.05). The interactions between group and frequency, between group and brain region, and between frequency and brain region were also not significant (*F*_(2,207)_ = 0.51, *P*>0.05; *F*_(2,207)_ = 0.91, *P*>0.05; *F*_(4,207)_ = 0.20, *P*>0.05). The interaction among group, frequency and brain region was not significant (*F*_(4,207)_ = 0.15, *P*>0.05).

**Fig 12:**
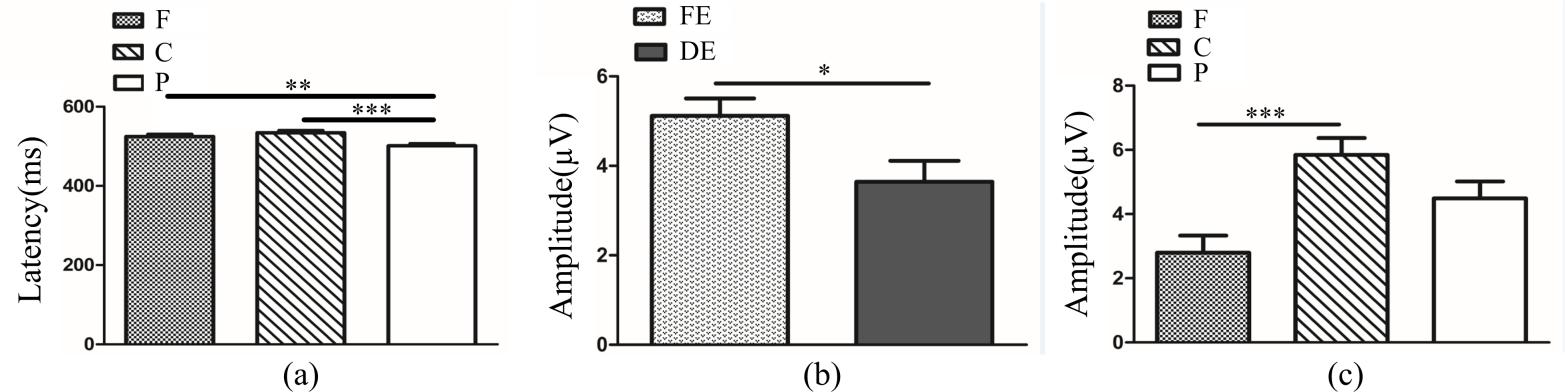
**(a)**. P3b latency in different brain regions in FE and DE (F vs. C *P*>0.05; F vs. P *P<*0.01; C vs. P *P<*0.001). **(b).** P3b amplitude between FE and DE (*P*<0.05). **(c)**. P3b amplitude in different brain regions in FE and DE (F vs. C *P*<0.001; F vs. P *P>*0.05; C vs. P *P*>0.05). The Y-axis represents the wave latency or amplitude range. Error bars indicate Standard Error (N_AA_ = 15 and N_NC_ = 10; * *P*<0.05; **P<0.01; ***P<0.001). FE: Fellow Eye; DE: Dominant Eye; F: Frontal Lobe; C: Central Lobe; P: Parietal Lobe; N_AA_: Number of Anisometopic Amblyopia; N_NC_: Number of Normal Control.

In fellow eye and dominant eye, we conducted a 2 (two groups: fellow eye and dominant eye) × 3 (frequencies: 3, 6 and 9 cpd) × 3 (brain regions: frontal, central and parietal lobe) ANOVA of latency and amplitude for P3b in target stimuli. Analysis of P3b latency showed a significant main effect of brain region (*F*_(2,207)_ = 10.36, *P<*0.001, η^2^ = 0.09). Multiple comparison tests using the Tukey HSD method showed that a longer latency occurred over the frontal lobe (524.93±5.30 ms) and central lobe (534.46±5.30 ms) compared with that over the parietal lobe (501.34±5.30 ms) (Fig 12A). The main effects of group and frequency were not significant (*F*_(1,207)_ = 3.18, *P*>0.05; *F*_(2,207)_ = 1.91, *P*>0.05). The interactions between group and frequency, between group and brain region, and between frequency and brain region were also not significant (*F*_(2,207)_ = 0.21, *P*>0.05; *F*_(2,207)_ = 0.54, *P*>0.05; *F*_(4,207)_ = 0.50, *P*>0.05). The interaction among group, frequency and brain region was not significant (*F*_(4,207)_ = 0.67, *P*>0.05). P3b amplitude analysis showed a significant main effect of group (*F*_(1,207)_ = 5.84, *P*<0.05, η^2^ = 0.03). Multiple comparison tests using the Tukey HSD method showed that the amplitude in the fellow eye (5.12±0.39 μV) was larger than that in the dominant eye (3.64±0.47 μV) (Fig 12B).The main effect of brain region was also significant (*F*_(2,207)_ = 8.26, *P*<0.001, η^2^ = 0.07). Multiple comparison tests using the Tukey HSD method showed that the amplitude over the central lobe (5.84±0.53 μV) was higher than in the frontal lobe (2.80±0.53 μV) and there was no difference between parietal regions (4.49±0.53 μV) and frontal/central lobe (Fig 12C). The main effect of frequency was not significant (*F*_(2,207)_ = 2.86, *P*>0.05). The interactions between group and frequency, between group and brain region, and between frequency and brain region were also not significant (*F*_(2,207)_ = 0.13, *P*>0.05; *F*_(2,207)_ = 0.66, *P*>0.05; *F*_(4,207)_ = 0.78, *P*>0.05). The interaction among group, frequency and brain region was not significant (*F*_(4,207)_ = 0.10, *P*>0.05).

**Fig 13:**
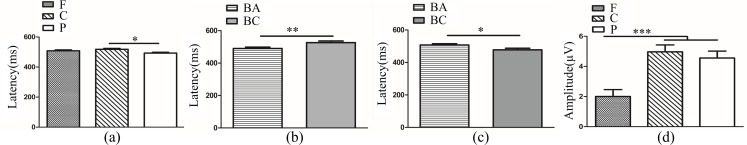
**(a)**. P3b latency in different brain regions in BA and BC (F vs. C *P*>0.05; F vs. P *P>*0.05; C vs. P *P<*0.05). **(b)**. P3b latency over frontal electrode between BA and BC (*P*<0.01). **(c)**. P3b latency over parietal electrode between BA and BC (*P*<0.05). **(d)**. P3b amplitude in different brain regions in BA and BC (F vs. C *P*<0.001; F vs. P *P<*0.001; C vs. P *P*>0.05). The Y-axis represents the wave latency or amplitude range. Error bars indicate Standard Error (N_AA_ = 15 and N_NC_ = 10; * *P*<0.05; **P<0.01; ***P<0.001). BA: Binoculus in Amblyopia, BC: Binoculus in Control; F: Frontal Lobe; C: Central Lobe; P: Parietal Lobe; N_AA_: Number of Anisometopic Amblyopia; N_NC_: Number of Normal Control.

In binoculus in amblyopia and binoculus in normal control, we conducted a 2 (two groups: binoculus in amblyopia and binoculus in normal control) × 3 (frequencies: 3, 6 and 9 cpd) × 3 (brain regions: frontal, central and parietal lobe) ANOVA of latency and amplitude for P3b in target stimuli. Analysis of P3b latency showed the main effect of brain region was significant (*F*_(2,207)_ = 4.98, *P*<0.01, η^2^ = 0.05). Multiple comparison tests using the Tukey HSD method showed that a longer latency occurred over the central lobe (518.31±5.82 ms) compared with that over the parietal lobe (492.57±5.82 ms) and there was no difference between frontal lobe (508.29±5.82 ms) and central/parietal lobe (Fig 13A). The interaction between group and brain region was significant (*F*_(2,207)_ = 7.97, *P*<0.001, η^2^ = 0.07). Simple-effects analysis showed that the difference in latency among the two groups was statistically significant over the frontal and parietal lobes. The P3b latency was shorter in the binoculus in amblyopia (490.25±8.04 ms) than that in normal control (526.33±9.85 ms) over the frontal lobe (Fig 13B). Over the central lobe, there was no difference between two groups (*t*_69_ = 0.71, *P*>0.05). Over the parietal region, the P3b latency was longer in the binoculus in amblyopia (507.27±8.06 ms) compared with binoculus in normal control (477.87±9.87 ms) (Fig 13C). The main effects of group and frequency were not significant (*F*_(1,207)_ = 0.51, *P*>0.05; *F*_(2,207)_ = 0.16, *P*>0.05). The interactions between group and frequency and between frequency and brain region were also not significant (*F*_(2,207)_ = 0.63, *P*>0.05; *F*_(4,207)_ = 0.16, *P*>0.05). The interaction among group, frequency and brain region was not significant (*F*_(4,207)_ = 0.22, *P*>0.05). P3b amplitude analysis showed a significant main effect of brain region (*F*_(2,207)_ = 12.50, *P*<0.001, η^2^ = 0.11). Multiple comparison tests using the Tukey HSD method showed that the amplitude over the central lobe (4.97±0.46 μV) and parietal lobe (4.53±0.46 μV) was higher than in the frontal lobe (2.00±0.46 μV) (Fig 13D). The main effects of group and frequency were not significant (*F*_(1,207)_ = 1.15, *P*>0.05; *F*_(2,207)_ = 0.06, *P*>0.05). The interactions between group and frequency, group and brain region and between frequency and brain region were also not significant (*F*_(2,207)_ = 0.09, *P*>0.05; *F*_(2,207)_ = 0.25, *P*>0.05;*F*_(4,207)_ = 0.15, *P*>0.05). The interaction among group, frequency and brain region was not significant (*F*_(4,207)_ = 0.42, *P*>0.05).

The results further revealed that RT and P3b latency in the amblyopic eye were longer than those in both eyes of amblyopia. The P3b amplitude was highest over the central and parietal regions. Therefore, the relationship between RT and P3b latency over the central and parietal regions was analyzed with the Pearson correlation method. However, there were no correlations between RT and P3b latency over the central and parietal regions (*r* = -0.06, *P>*0.05; *r* = 0.01, *P>*0.05).

We found that RT in amblyopic eye was longer than the binoculus in amblyopia and non-dominant eye of control. However, ACC was no significant difference in monocular eye and binocular eyes in amblyopia and normal control as well as the corresponding eyes between two groups. The novelty P300 amplitude was largest in amblyopic eye, followed by fellow eye, and smallest in binoculus in amblyopia. Besides, the novelty P300 amplitude in amblyopic and fellow eye was larger than binoculus in amblyopia over frontal lobe. Over central lobe, novelty P300 amplitude in amblyopic eye was largest, followed by fellow eye, and smallest in binoculus in amblyopia. Over parietal lobe, novelty P300 amplitude in amblyopic eye was larger than binoculus in amblyopia. Novelty P300 amplitude in amblyopic eye was larger than non-dominant eye. Novelty P300 amplitude in fellow eye was larger than dominant eye. The P3b latency was longer in amblyopic eye than fellow eye, binoculus in amblyopia and non-dominant eye of normal control. In addition, the ACC, RT, the latency and amplitude of novelty P300 and P3b were no differences in different eye conditions of normal controls.

## Discussion

The aim of this study was to investigate the processing of visual information about novelty stimuli and target stimuli in during monocular and binocular viewing conditions of amblyopia and normal control as well as the corresponding eyes of the two groups at different spatial frequencies.

Even though ACC, RT, and the latency and amplitude of novelty P300 and P3b showed no significant differences at different spatial frequencies in different eye conditions in amblyopia, normal control and the corresponding eyes of the two groups. However, we found that amblyopic eyes had longer RT, a longer latency of P3b and a larger amplitude of novelty P300 than binoculus in amblyopia and non-dominant eye of control. However, RT and P3b latencies over the central and parietal regions are independent. The largest P300 amplitude of novel stimuli was shown in the amblyopic eye, followed by the fellow eye and smallest in the binoculus of amblyopia. We could not find the effect of spatial frequencies in different eye conditions in amblyopia group and normal control group as well as the correspondings eye of the two groups. This may be that the spatial frequencies were 3,6 and 9cpd which all belonged to the medium frequencies. A range of frequencies, such as lower and higher one, would be considered to investigate the effect of different spatial frequencies on amblyopia in future study.

The behavioral experiments showed that the amblyopic eye took more time to respond when compared with both eyes of amblyopia and non-dominant eye of control. Although the fellow eye showed a markedly shortened RT compared with the amblyopic eye, no significant difference was found between them. This outcome is slightly different from that described in a previous study by Körtvélyes, Bankó [45], in which decreased ACC and longer RT in a gender categorization task were observed in the amblyopic eye when compared to the fellow eye and both eyes of amblyopia. This inconsistency may be due to amblyopic types, vision in the amblyopic eye, stimulus and task requirements. Bankó included patients with anisometropic and strabismic amblyopia, while we recruited only those with anisometropic amblyopia, allowing for the different mechanism of impairment in the two types of amblyopia [11, 46]. RT in different eye conditions is comparable in normal controls and this result may show that longer RT in amblyopic eye is not due to monocular condition when compared with binocular condition in amblyopia. There was no difference in ACC for different frequencies and eye conditions in anisometropic amblyopes and normal controls as well as the corresponding eyes. One possible explanation for this finding might be that the amblyopic eyes recruited even had 0.5 logMAR acuity or better vision, so that each eye in amblyopia and normal control could be qualified for the orientation discrimination tasks at these three spatial frequencies in our study. Larger samples and a range of spatial frequencies are needed to further address this hypothesis in future studies.

Some researchers have proposed that P3a is correlated with the selection of stimulus information regulated by attentional orienting [28, 29], which reflects the shift of previous attentional focus to attentional processes toward the infrequent stimulus [47]. Our study found no difference in novelty P300 latency in three eye conditions in amblyopia and normal control as well as the corresponding eyes of the two groups, which may have been because the speed of selecting stimulus information was comparable in different eye conditions in amblyopia and normal control as well as the corresponding eyes of the two groups.

Novelty P300 is a kind of P3a, the amplitude of which is determined by the amount of focal attention [48]. Increased perceptual discrimination difficulty between the target and standard stimuli can increase P3a amplitude. P3a also could be elicited by infrequent disruption of target/standard discrimination task regardless of whether the distracting stimuli was novel or not, when the sufficient target/standard discrimination was difficult. The reason may be that the engaged distracter processing was stronger for the harder tasks, so P3a amplitude was enhanced by increasing focal attention constraining resource allocation operations[49, 50]. We found that novelty P300 amplitude was largest in the amblyopic eye, followed by the fellow eye, and smallest in the binoculus of amblyopia. Although the difference in the three eye conditions showed some discrepancy in different brain regions, all results were consistent in that the amplitude of novelty P300 in the amblyopic eye was larger than that in the binoculus of amblyopia but there was no difference in three eye conditions of normal controls. We also found that novelty P300 amplitude was larger in the amblyopic eye than non-dominant eye. These results might be because the task is more difficult for the amblyopic eye, so it needs to integrate more focal attention to select the stimuli information. Furthermore, binoculus in amblyopia need less attentional resources to process visual information than the fellow eye but the attentional resources are equal different eye conditions in normal controls. It may imply that the fellow eye also needs more attentional resources to process visual information than both eyes. Previous studies have found that the fellow eye in amblyopia also suffers perceptual deficits [51, 52]. The difficulty of discrimination tasks in simultaneous binocular input condition in amblyopia was reduced due to facilitating effect so it is not necessary to integrate more attention to select stimuli information in this condition. We propose that the amblyopic eye facilitates the cognitive processing of the fellow eye instead of being suppressed in the binocular viewing condition. This may be because many anisometropic amblyopes retain some stereopsis [8]. Generally, worse visual acuity is associated with worse stereoacuity in anisometropic amblyopes. The amblyopic eyes we recruited had 0.5 logMAR acuity or better acuity, and all anisometropic amblyopes passed the Randot circle test. Indeed, this may be the reason why the amblyopic eye was not completely suppressed in a binocular viewing condition. Meanwhile, the novelty P300 amplitude is larger in fellow eye than that in dominant eye. This may imply that the same discrimination task is more difficult for the fellow eye, so it needs to integrate more focal attention to select the stimuli information even though the acuity between the corresponding eyes of two groups is comparative. The result also suggests that the fellow eye in amblyopia suffers perceptual deficits as discussed before. The latency and amplitude of novelty P300 were not different in different eye condition of normal control and the binoculus between the two groups. These results indicate that each eye condition in normal control group and binocular viewing condition between two groups have the equivalent speed and capacity to process novel stimuli. Novelty P300 amplitude is larger over the central and parietal region than the frontal region. In contrast to previous findings, however, we could not detect the frontal maximum. The reason for this is not clear but it may have something to do with the orientation task. Many studies have shown that posterior brain regions are more sensitive to orientation [53–55]. Additional studies are needed to confirm whether the sensitivity of orientation perception over different brain regions affects the amplitude distribution of novelty P300.

P3b is thought to reflect neuronal activity associated with revision of the working memory within the stimulus environment [17, 56]. P3b amplitude and latency, linked to various attentional and memory processes, are measures of processing capacity and speed [27, 29]. P3b latency represents the speed of stimuli evaluation and classification and varies with individual differences in cognitive capability [48]. We found that P3b latency was longer in the amblyopic eye than in the fellow eye, binoculus in amblyopia and non-dominant eye of control. The reason for this finding may be that the amblyopic eye has abnormal cognitive capability, so that it must spend more time in evaluating and classifying stimuli in the same discrimination task. P3b amplitude is related to the amount of attentional resources allocated to a stimulus [57, 58]. However, we did not find that P3b amplitude was different among three viewing conditions of amblyopia and normal control. It is possible that the amblyopic eye has a compensated ability that relies on spending more time in allocating comparative attention resources to complete the same tasks. We found that P3b amplitude in fellow eye was larger than dominant eye. It may indicate that fellow eye needs to allocate more attentional resources to the same difficult discrimination task. Even though P3b latency was not different between the binoculus of two groups, the differences existed over the frontal and parietal area. It is very interesting that shorter latency is in the binoculus of amblyopia over the frontal area but opposite over the parietal area. According to previous studies, the maximum amplitude of P3b is over the parietal area [35]. Longer P3b latency in binoculus of amblyopia may imply that the binoculus in amblyopia is slower to process the target stimuli but the shorter latency over the frontal area may be a kind of compensated mechanism. The different eye conditions of normal control are comparable to deal with the target stimuli so that there were no differences in latency and amplitude of P3b. Our research showed that the amplitude over the central and/or the parietal lobes was larger than that in the frontal area, which is consistent with previous studies [41, 48].

The outcomes between novelty P300 and P3b were inconsistent in our study, the reason for this may be that these factors are governed by different mechanisms [59, 60]. P3b, having a stronger parietal contribution, reflects target detection in a bottom-up mechanism, while P3a, having a stronger frontal contribution, reflects target detection in a top-down mechanism.

Our study did not find that the extent of neural responses to the visual information of different spatial frequencies from the amblyopia and normal controls were different during monocular and binocular viewing conditions as well as the corresponding eyes of two groups. However, the amblyopic eye showed slower speed for evaluation and classification in comparison to binoculus in amblyopia and non-dominant eye of normal control. One possible explanation for this finding might be that the amblyopic eye has a slower speed of processing for information in whole spatial frequencies used in the study.

The latency of N2 in novel stimuli and target stimuli showed no significant difference in different eye conditions in amblyopia and normal control as well as the corresponding eyes of two groups. This result indicates that the longer latency of P3b in the amblyopic eye was not due to prolonged latency of the previous component.

We found that there was no correlation between RT and P3b latency over the parietal and central regions. Thus, P300 latency is independent on behavioral RT, and this result is similar to those reported in previous studies [38, 56, 61]. This finding might be because P300 latency is considered as a measure of stimulus classification speed which is independent of response selection processes and as an index of the processing time required before response generation. These advantages make it a valuable tool to assess cognitive function [62].

Some studies have indicated that P3b latency in Cz and /or Pz is related to the dopamine level [63, 64]. Stanzione, Fattapposta (64] found that P3b latency of Parkinson’s patients increased before therapy and recovered during dopaminergic monotherapy, while that of healthy subjects with the same therapy did not decrease. This result may show a possible dopaminergic component of P3b origin. Indeed, treatment with levodopa/carbidopa can improve and maintain the visual acuity of amblyopic patients [65, 66]. Further studies are needed to confirm whether levodopa/carbidopa therapy in patients with amblyopia can also shorten the latency of P3b. Perhaps this approach may be used to evaluate the efficacy of levodopa/carbidopa on amblyopes once this hypothesis is confirmed.

Seppänen, Pesonen [67] proposed that shortened P3b latency was presented after auditory perceptual learning. These authors considered that short-term P3b plasticity could be enhanced depending on music training-induced changes in attentional skills. A good deal of evidence suggests that visual perceptual learning is a promising way to alter the neural balance in amblyopes by increasing the signal, reducing noise, or modulating attention beyond the critical period of visual development [68, 69]. However, it remains unknown whether visual perceptual learning would also shorten the P3b latency in amblyopia. Hence, further studies are necessary to investigate this hypothesis; if confirmed, this method may be used to investigate the mechanism of perceptual learning in amblyopia.

In future study, other discrimination tasks will be used that may be important to decipher what mechanisms/functions are affected in amblyopoia. Meanwhile, it is worth investigating whether neural process is different between subjects with right and left amblyopic eye in the next study.

## Limitation

Some limitations should be considered in interpreting our findings. First, the sample size was small, and larger sample studies are needed to confirm our hypothesis. Second, the study population was limited to anisometropic amblyopia, so future studies need to be extended to other types of amblyopia, such as strabismic amblyopia, to investigate and compare their neural mechanisms. Finally, we cannot avoid some unmeasured and unknown confounders that may have affected the results.

## Conclusion

In conclusion, our findings suggest that the amblyopic eye and fellow eye are abnormal at the middle and late stages of cognitive processing, reflected by the longer latency of P3b components as well as larger amplitude of novelty P300 and P3b. This result implies the abnormality of cognitive capacity in the amblyopic and fellow eye. In addition, our results show that cognitive abnormality does not vary with different spatial frequencies for mild to moderate anisometropic amblyopes. However, the facilitation is generated to simplify cognitive processing in anisometropic amblyopes during binocular information input, indicating that the amblyopic eye promotes the cognitive processing of the fellow eye instead of being completely suppressed. The cognitive processing of orientation discrimination task is comparable in different eye conditions of normal control at different spatial frequencies.

## Supporting information

S1 Data The accuracy and reaction time of different eye conditions at three spatial frequencies in anisometropic amblyopia and normal control. Group 1: Anisometropic Amblyopes; Group 2: Normal Controls; AE: Amblyopic Eye; FE: Fellow eye; BA: Binoculus in Amblyopia; ND: Non-dominant Eye; DE: Dominant Eye; BC: Binoculus in Control.
(XLSX)

S2 Data Novelty stimuli-N2 latency. Group 1: Anisometropic Amblyopes; Group 2: Normal Controls; AE: Amblyopic Eye; FE: Fellow eye; BA: Binoculus in Amblyopia; ND: Non-dominant Eye; DE: Dominant Eye; BC: Binoculus in Control; F: Frontal Electrode; C: Central Electrode; P: Parietal Electrode.
(XLSX)

S3 Data The latency and amplitude of novelty P300 in novelty stimuli. Group 1: Anisometropic Amblyopes; Group 2: Normal Controls; AE: Amblyopic Eye; FE: Fellow eye; BA: Binoculus in Amblyopia; ND: Non-dominant Eye; DE: Dominant Eye; BC: Binoculus in Control; F: Frontal Electrode; C: Central Electrode; P: Parietal Electrode.
(XLSX)

S4 Data Target stimuli-N2 latency. Group 1: Anisometropic Amblyopes; Group 2: Normal Controls; AE: Amblyopic Eye; FE: Fellow eye; BA: Binoculus in Amblyopia; ND: Non-dominant Eye; DE: Dominant Eye; BC: Binoculus in Control; F: Frontal Electrode; C: Central Electrode; P: Parietal Electrode.
(XLSX)

S5 Data The latency and amplitude of P3b in target stimuli. Group 1: Anisometropic Amblyopes; Group 2: Normal Controls; AE: Amblyopic Eye; FE: Fellow eye; BA: Binoculus in Amblyopia; ND: Non-dominant Eye; DE: Dominant Eye; BC: Binoculus in Control; F: Frontal Electrode; C: Central Electrode; P: Parietal Electrode.
(XLSX)

## References

[1] Birch EE. Amblyopia and Binocular Vision. Progress in Retinal & Eye Research. 2012;33(1):67–84. 23201436 10.1016/j.preteyeres.2012.11.001 PMC3577063

[2] Hamm LM, Black J, Dai S, Thompson B. Global processing in amblyopia: a review. Frontiers in Psychology. 2014;5(7):583. 24987383 10.3389/fpsyg.2014.00583 PMC4060804

[3] Barrett BT, Bradley A, Candy TR. The relationship between anisometropia and amblyopia. Progress in retinal and eye research. 2013;36:120–58. doi: 10.1016/j.preteyeres.2013.05.001. ; PubMed Central PMCID: PMC3773531. 23773832 10.1016/j.preteyeres.2013.05.001 PMC3773531

[4] Bonneh YS, Sagi D, Polat U. Local and non-local deficits in amblyopia: acuity and spatial interactions. Vision Research. 2005;44(27):3099–110. 10.1016/j.visres.2004.07.031 15482798

[5] Holmes JM, Clarke MP. Amblyopia. Lancet. 2006;367(9519):1343–51. doi: 10.1016/S0140-6736(06)68581-4 16631913 10.1016/S0140-6736(06)68581-4

[6] McKee SP, Levi DM, Movshon JA. The pattern of visual deficits in amblyopia. J Vis. 2003;3(5):5-. 10.1167/3.5.5 12875634

[7] Ding J, Klein SA, Levi DM. Binocular combination in abnormal binocular vision. J Vis. 2013;13(2):14 doi: 10.1167/13.2.14. ; PubMed Central PMCID: PMC4521338. 23397039 10.1167/13.2.14 PMC4521338

[8] Levi DM, Knill DC, Bavelier D. Stereopsis and amblyopia: a mini-review. Vision research. 2015;114:17–30. doi: 10.1016/j.visres.2015.01.002. ; PubMed Central PMCID: PMC4519435. 25637854 10.1016/j.visres.2015.01.002 PMC4519435

[9] Levi DM, Hariharan S, Klein SA. Suppressive and facilitatory spatial interactions in amblyopic vision. Vision research. 2002;42(11):1379–94. 12044744 10.1016/s0042-6989(02)00061-5

[10] Joly O, Franko E. Neuroimaging of amblyopia and binocular vision: a review. Frontiers in integrative neuroscience. 2014;8:62 doi: 10.3389/fnint.2014.00062. ; PubMed Central PMCID: PMC4123726. 25147511 10.3389/fnint.2014.00062 PMC4123726

[11] Bankó ÉM, Körtvélyes J, Németh J, Weiss B, Vidnyánszky Z. Amblyopic deficits in the timing and strength of visual cortical responses to faces. Cortex. 2013;49(4):1013–24. doi: 10.1016/j.cortex.2012.03.021 22578711 10.1016/j.cortex.2012.03.021

[12] Banko EM, Körtvélyes J, Weiss B, Vidnyanszky Z. How the visual cortex handles stimulus noise: insights from amblyopia. PloS one. 2013;8(6):e66583 doi: 10.1371/journal.pone.0066583 23818947 10.1371/journal.pone.0066583 PMC3688592

[13] Hou C, Pettet MW, Norcia AM. Abnormalities of coherent motion processing in strabismic amblyopia: visual-evoked potential measurements. J Vis. 2008;8(4):2-. doi: 10.1167/8.4.2 18484841 10.1167/8.4.2 PMC4386923

[14] Parisi V, Scarale ME, Balducci N, Fresina M, Campos EC. Electrophysiological detection of delayed postretinal neural conduction in human amblyopia. Invest Ophthalmol Vis Sci. 2010;51(10):5041–8. doi: 10.1167/iovs.10-5412 20463312 10.1167/iovs.10-5412

[15] Awh E, Anllo-Vento L, Hillyard SA. The Role of Spatial Selective Attention in Working Memory for Locations: Evidence from Event-Related Potentials. Journal of Cognitive Neuroscience. 2000;12(5):840–7. 11054925 10.1162/089892900562444

[16] Vorkapić ST, Tadinac M, Kulenović A. Event-related potentials and abstract thinking. Dani Ramira I Zorana Bujasa. 2007.

[17] Donchin E, Coles MG. Is the P300 component a manifestation of context updating. Behav Brain Sci. 1988;11(3):357–427.

[18] Polich J. P300 clinical utility and control of variability. J Clin Neurophysiol. 1998;15(1):14–33. 9502510 10.1097/00004691-199801000-00004

[19] Polich J, Herbst KL. P300 as a clinical assay: rationale, evaluation, and findings. Int J Psychophysiol. 2000;38(1):3–19. 11027791 10.1016/s0167-8760(00)00127-6

[20] Dejanovic M, Ivetic V, Nestorovic V, Milanovic Z, Eric M. The value of P300 Event Related Potentials in the assessment of cognitive function in subclinical hypothyroidism. Minerva Endocrinol. 2015:In press. PubMed doi: 10.23736/S0391-1977.16.02327-0 . 26337490 10.23736/S0391-1977.16.02327-0

[21] Jimenez-Rodriguez A, Rodriguez-Sotelo JL, Osorio-Forero A, Medina JM, de Mejia FR. The shape of dementia: new measures of morphological complexity in event-related potentials (ERP) and its application to the detection of Alzheimer's disease. Med Biol Eng Comput. 2015;53(9):889–97. doi: 10.1007/s11517-015-1283-x. . 25868458 10.1007/s11517-015-1283-x

[22] Kirkil G, Tug T, Ozel E, Bulut S, Tekatas A, Muz MH. The evaluation of cognitive functions with P300 test for chronic obstructive pulmonary disease patients in attack and stable period. Clin Neurol Neurosurg. 2007;109(7):553–60. doi: 10.1016/j.clineuro.2007.03.013 17532116 10.1016/j.clineuro.2007.03.013

[23] Hamm LM, Black J, Dai S, Thompson B. Global processing in amblyopia: a review. Front Psychol. 2014;5:583 doi: 10.3389/fpsyg.2014.00583. ; PubMed Central PMCID: PMCPMC4060804. 24987383 10.3389/fpsyg.2014.00583 PMC4060804

[24] Joshi M, Simmers A, Jeon S. Deficits in integration of global motion and form in noise is associated with the severity and type of amblyopia. J Vis. 2015;15(12):193-.

[25] Ho CS, Giaschi DE. Low- and high-level motion perception deficits in anisometropic and strabismic amblyopia: evidence from fMRI. Vision research. 2009;49(24):2891–901. Epub 2009/08/01. doi: 10.1016/j.visres.2009.07.012. . 19643122 10.1016/j.visres.2009.07.012

[26] Husk JS, Farivar R, Hess RF. Amblyopic deficits in processing structure-from-motion. J Vis. 2012;12(4). Epub 2012/04/17. doi: 10.1167/12.4.4. . 22505619 10.1167/12.4.4

[27] Kok A. Event-related-potential (ERP) reflections of mental resources: a review and synthesis. Biol Psychol. 1997;45(1):19–56. 9083643 10.1016/s0301-0511(96)05221-0

[28] Kok A. On the utility of P3 amplitude as a measure of processing capacity. Psychophysiology. 2001;38(3):557–77. 11352145 10.1017/s0048577201990559

[29] Rushby JA, Barry RJ, Doherty RJ. Separation of the components of the late positive complex in an ERP dishabituation paradigm. Clinical neurophysiology: official journal of the International Federation of Clinical Neurophysiology. 2005;116(10):2363–80. doi: 10.1016/j.clinph.2005.06.008. . 16099212 10.1016/j.clinph.2005.06.008

[30] Guo Q, Tang Y, Li H, Zhang T, Li J, Sheng J, et al Both volumetry and functional connectivity of Heschl's gyrus are associated with auditory P300 in first episode schizophrenia. Schizophr Res. 2014;160(1):57–66. 25458859 10.1016/j.schres.2014.10.006

[31] Inoue Y, Inagaki M, Gunji A, Kokubo N, Kaga M. [Cerebral inhibitory functioning in patients with attention deficit/hyperactivity disorder. I. Analysis of non-target-P300 component in a visual oddball paradigm]. No To Hattatsu. 2007;39(4):263–7. 17633082

[32] Polich J. Overview of P3a and P3b In: Polich J, editor. Detection of change: event-related potential and fmri findings. Norwell: Kluwer Academic Press; 2003 p. 83–98.

[33] Friedman D, Simpson GV. ERP amplitude and scalp distribution to target and novel events: effects of temporal order in young, middle-aged and older adults. Brain Res Cogn Brain Res. 1994;2(1):49–63. 7812178 10.1016/0926-6410(94)90020-5

[34] Knight RT. Contribution of human hippocampal region to novelty detection. Nature. 1996;383(6597):256–9. doi: 10.1038/383256a0 8805701 10.1038/383256a0

[35] Johnson R. On the neural generators of the P300 component of the event‐related potential. Psychophysiology. 1993;30(1):90–7. 8416066 10.1111/j.1469-8986.1993.tb03208.x

[36] Polich J, Heine MR. P300 topography and modality effects from a single‐stimulus paradigm. Psychophysiology. 1996;33(6):747–52. 8961797 10.1111/j.1469-8986.1996.tb02371.x

[37] Duncan-Johnson CC. P3 latency: a new metric of information processing. Psychophysiology. 1981;18:207–15. 7291436 10.1111/j.1469-8986.1981.tb03020.x

[38] Verleger R. On the utility of P3 latency as an index of mental chronometry. Psychophysiology. 1997;34(2):131–56. 9090263 10.1111/j.1469-8986.1997.tb02125.x

[39] Kutas M, McCarthy G, Donchin E. Augmenting mental chronometry: the P300 as a measure of stimulus evaluation time. Science. 1977;197(4305):792–5. 887923 10.1126/science.887923

[40] Ila AB, Polich J. P300 and response time from a manual Stroop task. Clinical neurophysiology: official journal of the International Federation of Clinical Neurophysiology. 1999;110(2):367–73. 10210626 10.1016/s0168-5597(98)00053-7

[41] Pontifex MB, Hillman CH, Polich J. Age, physical fitness, and attention: P3a and P3b. Psychophysiology. 2009;46(2):379–87. doi: 10.1111/j.1469-8986.2008.00782.x 19170947 10.1111/j.1469-8986.2008.00782.x PMC2763440

[42] Dolman CP. Tests for Determining the Sighting Eye. American Journal of Ophthalmology. 1919;2(12):867-.

[43] Samarawickrama C, Wang JJ, Huynh SC, Wang XY, Burlutsky G, Stapleton F, et al Macular thickness, retinal thickness, and optic disk parameters in dominant compared with nondominant eyes. J AAPOS. 2009;13(2):142–7. doi: 10.1016/j.jaapos.2008.11.004 19393511 10.1016/j.jaapos.2008.11.004

[44] Bryden MP. Measuring handedness with questionnaires. Neuropsychologia. 1977;15(4–5):617–24. 896019 10.1016/0028-3932(77)90067-7

[45] Körtvélyes J, Bankó É, Andics A, Rudas G, Németh J, Hermann P, et al Visual cortical responses to the input from the amblyopic eye are suppressed during binocular viewing. Acta Biologica Hungarica. 2012;63(Supplement 1):65–79. 22453742 10.1556/ABiol.63.2012.Suppl.1.7

[46] Wang X, Cui D, Zheng L, Yang X, Yang H, Zeng J. Combination of blood oxygen level–dependent functional magnetic resonance imaging and visual evoked potential recordings for abnormal visual cortex in two types of amblyopia. Mol Vis. 2012;18:909–19. 22539870 PMC3335782

[47] Squires NK, Squires KC, Hillyard SA. Two varieties of long-latency positive waves evoked by unpredictable auditory stimuli in man. Electroencephalogr Clin Neurophysiol. 1975;38(4):387–401. 46819 10.1016/0013-4694(75)90263-1

[48] Polich J. Updating P300: an integrative theory of P3a and P3b. Clinical neurophysiology: official journal of the International Federation of Clinical Neurophysiology. 2007;118(10):2128–48. 17573239 10.1016/j.clinph.2007.04.019 PMC2715154

[49] Demiralp T, Ademoglu A, Comerchero M, Polich J. Wavelet analysis of P3a and P3b. Brain topography. 2001;13(4):251–67. 11545154 10.1023/a:1011102628306

[50] Polich J, Comerchero MD. P3a from Visual Stimuli: Typicality, Task, and Topography. Brain Topography. 2003;15(3):141–52. 12705810 10.1023/a:1022637732495

[51] Ho CS, Giaschi DE, Boden C, Dougherty R, Cline R, Lyons C. Deficient motion perception in the fellow eye of amblyopic children. Vision research. 2005;45(12):1615–27. doi: 10.1016/j.visres.2004.12.009 15781077 10.1016/j.visres.2004.12.009

[52] Meier K, Sum B, Giaschi D. Global motion perception deficits in children with amblyopia as a function of spatial and temporal stimulus parameters. J Vis. 2015;15(12):653-. 10.1016/j.visres.2016.06.011 27426263

[53] Zhou B, Yang S, Mao L, Han S. Visual feature processing in the early visual cortex affects duration perception. Journal of experimental psychology General. 2014;143(5):1893–902. Epub 2014/07/08. doi: 10.1037/a0037294. . 25000445 10.1037/a0037294

[54] Schummers J, Sharma J, Sur M. Bottom-up and top-down dynamics in visual cortex. Progress in brain research. 2005;149:65–81. Epub 2005/10/18. doi: 10.1016/S0079-6123(05)49006-8. . 16226577 10.1016/S0079-6123(05)49006-8

[55] Wilson DE, Whitney DE, Scholl B, Fitzpatrick D. Orientation selectivity and the functional clustering of synaptic inputs in primary visual cortex. Nature neuroscience. 2016;19(8):1003–9. Epub 2016/06/14. doi: 10.1038/nn.4323. . 27294510 10.1038/nn.4323 PMC5240628

[56] Donchin E. Surprise!… surprise? Psychophysiology. 1981;18(5):493–513. 7280146 10.1111/j.1469-8986.1981.tb01815.x

[57] Polich J. Task difficulty, probability, and inter-stimulus interval as determinants of P300 from auditory stimuli. Electroencephalogr Clin Neurophysiol. 1987;68(4):311–20. 2439311 10.1016/0168-5597(87)90052-9

[58] Polich J. Meta‐analysis of P300 normative aging studies. Psychophysiology. 1996;33(4):334–53. 8753933 10.1111/j.1469-8986.1996.tb01058.x

[59] Bledowski C, Prvulovic D, Goebel R, Zanella FE, Linden DE. Attentional systems in target and distractor processing: a combined ERP and fMRI study. NeuroImage. 2004;22(2):530–40. doi: 10.1016/j.neuroimage.2003.12.034. . 15193581 10.1016/j.neuroimage.2003.12.034

[60] Buschman TJ, Miller EK. Top-down versus bottom-up control of attention in the prefrontal and posterior parietal cortices. Science. 2007;315(5820):1860–2. doi: 10.1126/science.1138071 17395832 10.1126/science.1138071

[61] Giedke H, Thier P, Bolz J. The relationship between P 3-latency and reaction time in depression. Biol Psychol. 1981;13:31–49. 7343001 10.1016/0301-0511(81)90026-0

[62] Vecchio F, Maatta S. The use of auditory event-related potentials in Alzheimer's disease diagnosis. International journal of Alzheimer's disease. 2011;2011:653173 doi: 10.4061/2011/653173. ; PubMed Central PMCID: PMC3100636. 21629759 10.4061/2011/653173 PMC3100636

[63] Hansenne M, Pitchot W, Moreno AG, Papart P, Timsit-Berthier M, Ansseau M. Catecholaminergic function and P300 amplitude in major depressive disorder (P300 and catecholamines). Electroencephalogr Clin Neurophysiol. 1995;96(2):194–6. 7535224 10.1016/0168-5597(94)00317-8

[64] Stanzione P, Fattapposta F, Giunti P, D'Alessio C, Tagliati M, Affricano C, et al P300 variations in parkinsonian patients before and during dopaminergic monotherapy: a suggested dopamine component in P300. Electroencephalogr Clin Neurophysiol. 1991;80(5):446–53. 1716570 10.1016/0168-5597(91)90093-d

[65] Dadeya S, Vats P, Malik K. Levodopa/carbidopa in the treatment of amblyopia. J Pediatr Ophthalmol Strabismus. 2009;46(2):87–90. 19343970 10.3928/01913913-20090301-07

[66] Rashad MA. Pharmacological enhancement of treatment for amblyopia. Clin Ophthalmol. 2012;6:409 doi: 10.2147/OPTH.S29941 22536029 10.2147/OPTH.S29941 PMC3334227

[67] Seppänen M, Pesonen A-K, Tervaniemi M. Music training enhances the rapid plasticity of P3a/P3b event-related brain potentials for unattended and attended target sounds. Atten Percept Psychophys. 2012;74(3):600–12. doi: 10.3758/s13414-011-0257-9 22222306 10.3758/s13414-011-0257-9

[68] Hussain Z, Astle AT, Webb BS, McGraw PV. The challenges of developing a contrast-based video game for treatment of amblyopia. Front Psychol. 2014;5:1210 doi: 10.3389/fpsyg.2014.01210. ; PubMed Central PMCID: PMC4217344. 25404922 10.3389/fpsyg.2014.01210 PMC4217344

[69] Tsirlin I, Colpa L, Goltz H, Wong A. Plasticity in adult amblyopia: a meta-review and analysis. J Vis. 2015;15(12):656-. 10.1167/iovs.15-16583 26114483

